# Metal–Organic Frameworks Constructed from Branched Oligomers

**DOI:** 10.1021/acs.inorgchem.3c03452

**Published:** 2024-01-12

**Authors:** Hyunyong Kim, Seth M. Cohen

**Affiliations:** † Department of Chemistry and Biochemistry, University of California, San Diego, La Jolla, California 92093, United States

## Abstract

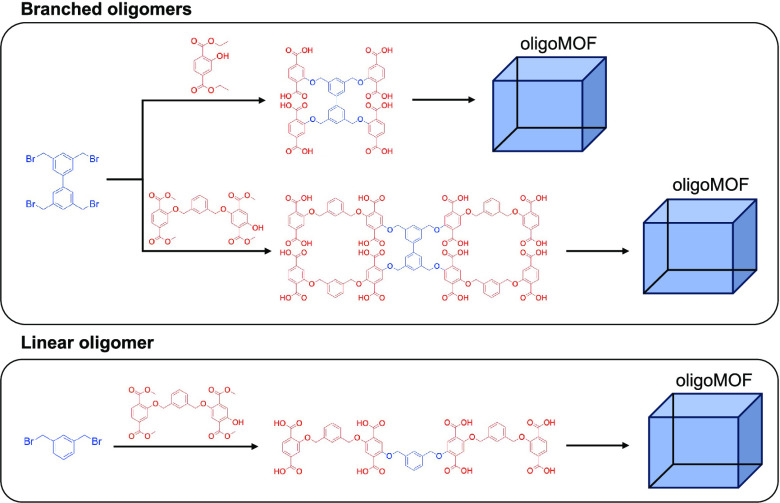

Metal–organic frameworks (MOFs) prepared from oligomeric or polymeric organic ligands have been studied and are termed oligoMOFs and polyMOFs, respectively. Herein, several oligoMOFs are described that have been prepared from branched oligomers with dendritic or star-like architectures. Branched oligomeric ligands with four (**4(H**_**2**_**bdc)-b**) or eight (**8(H**_**2**_**bdc)-b**) 1,4-benzene dicarboxylic acid (H_2_bdc) groups were prepared and used to synthesize isoreticular-type Zn(II)-based MOFs (IRMOF). A branched tetramer (**4(H**_**2**_**bdc)-b**) produced an oligoIRMOF-1 with improved ambient stability compared with IRMOF-1 or previously described oligoMOFs. To understand the effect of the ligand architecture, oligoIRMOFs were also prepared from a linear tetramer (**4(H**_**2**_**bdc)-l**). For a branched octamer (**8(H**_**2**_**bdc)-b**), it was found that the addition of an organic base was required to produce crystalline oligoIRMOFs. Multivariate MOFs (MTV-MOFs) could also be readily prepared with a combination of an octamer (**8(H**_**2**_**bdc)-b**) and H_2_bdc.

## Abstract 2

Metal–organic frameworks (MOFs) prepared from oligomeric or polymeric organic ligands have been studied and are termed oligoMOFs and polyMOFs, respectively.

Metal–organic frameworks (MOFs) are crystalline materials consisting of organic linkers and metal clusters.^1^ The tunable structure and porosity of MOFs have made these versatile materials attractive for a variety of applications,^2^ such as separations,^3^ catalysis,^4^ and sensors.^5^ The properties of MOFs can be tuned via various methods, including the choice of the organic linkers.^6^

The use of oligomeric and polymeric ligands (as opposed to typical molecular, monomeric ligands) has been reported for the synthesis of oligoMOFs and polyMOFs.^7−10^ Polymer chain length and molecular weight can affect the morphology and properties of the resulting polyMOFs.^10^ Oligomeric MOFs (oligoMOFs) have been proposed as intermediate species to understand the chemical space between conventional MOFs and polyMOFs. Oligomeric ligands consisting of two or three 1,4-benzene dicarboxylic acid (H_2_bdc) units connected by alkyl or xylyl tethers have been utilized to synthesize isoreticular metal–organic framework-1 (oligoIRMOF-1) derivatives.^11,12^ The crystallinity, porosity, and stability of previously reported oligoMOFs were dependent on the length of the alkyl chains in the ligand.

Branched ligands with multiple carboxylate groups are commonly used building blocks for MOF synthesis, with variations in geometry, rigidity, and the position of donor groups (e.g., carboxylates).^13^ MOFs derived from such branched ligands have demonstrated important characteristics compared to those derived from simple linear organic ligands (e.g., H_2_bdc). For example, the tritopic ligand (1,3,5-benzene tribenzoic acid) combined with Zn(II) was used to form one of the first microporous MOFs (MOF-177) displaying high carbon dioxide^14^ and hydrogen uptake.^15,16^ Similarly, 1,3,6,8-tetrakis[*p*-benzoic acid]pyrene was utilized to construct the Zr(IV)-based mesoporous NU-1000. The high stability and large pores of NU-1000 have made this MOF widely studied for the utilization of selective adsorption of carbohydrates,^17^ removal of atrazine,^18^ catalytic hydrolysis of phosphoesters,^19^ and oxidative dehydrogenation of propane.^20^ A highly branched hexacarboxylic linker with triazole groups (5,5′,5″-(4,4′,4″-(benzene-1,3,5-triyl)tris(1*H*-1,2,3-triazole-4,1-diyl))triisophtalic acid) was used to construct Cu(II)-based NU-125, which was studied as an absorbent for methane^21^ and oxygen.^22^ Taking inspiration from the use of branched molecular ligands for the formation of MOFs, as well as branching introduced in dendritic and star polymers, the use of branched oligomeric ligands is reported here for the formation of oligoMOFs. Both tetramer (**4(H**_**2**_**bdc)-b**) and octamer (**8(H**_**2**_**bdc)-b**) ligands were prepared containing four and eight H_2_bdc units, respectively (Scheme 1). In addition, a linear tetramer (**4(H**_**2**_**bdc)-l**) was prepared to compare the characteristics of branched versus linear ligands, both during oligoMOF synthesis and in the resulting material properties.

**Scheme 1:**
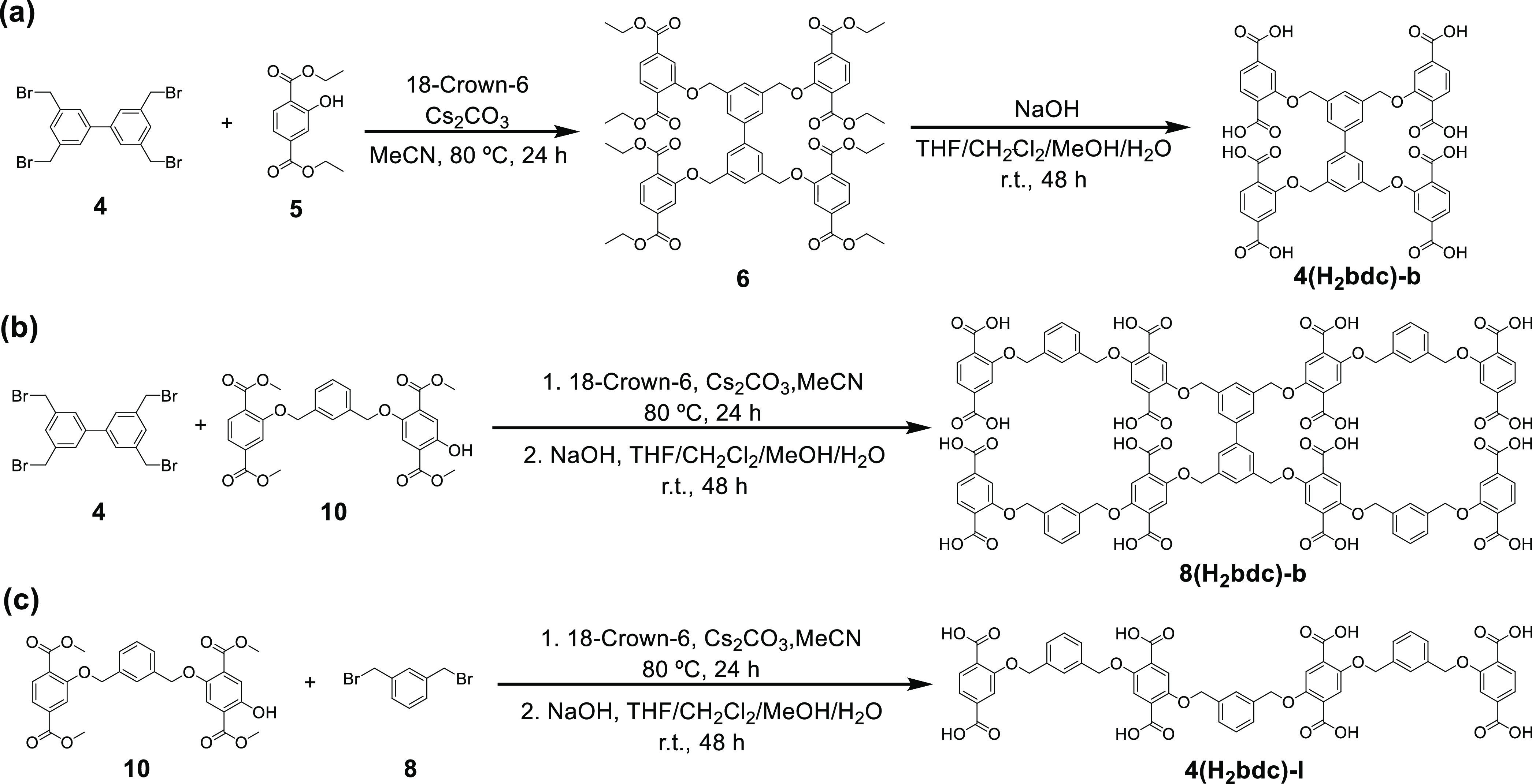
Synthetic Scheme of Functionalized Ligands: (a) Branched Tetramer (**4(H_2_bdc)-b**), (b) Branched Octamer (**8(H_2_bdc)-b**), and (c) Linear Tetramer (**4(H_2_bdc)-l**) Architectures.

A branched tetrameric oligomer (**4(H**_**2**_**bdc)-b**) was prepared to explore the structure and properties of the resulting oligoMOFs (Scheme 1a). The reaction between a biphenyl core with four bromomethyl units (**4**) and diethyl 2-hydroxyterephtalate (**5**) in the presence of cesium carbonate as a base and 18-crown-6 as a phase transfer catalyst gave the desired branched tetrameric oligomer in an ester protected form (**6**). Deprotection of the ester groups was performed by adding an excess of sodium hydroxide to give the branched tetramer with four H_2_bdc groups (**4(H**_**2**_**bdc)-b**) in quantitative yield.

For preparation of an oligoIRMOF from **4(H**_**2**_**bdc)-b**, a variety of reaction conditions were explored, including different solvents (dimethylformamide, DMF; diethylformamide, DEF) and temperatures (80, 100 °C) (Figures S4 and S5). Using DEF as the solvent and heating at 100 °C with slow ramping (0.5 °C/min) gave oligoMOFs with good crystallinity and porosity (Figure 1). Activated oligoIRMOF-1-4(bdc)-b gave a Brunauer–Emmett–Teller (BET) surface area of 1817 ± 13 m^2^/g (Figure 1b).

**Figure 1:**
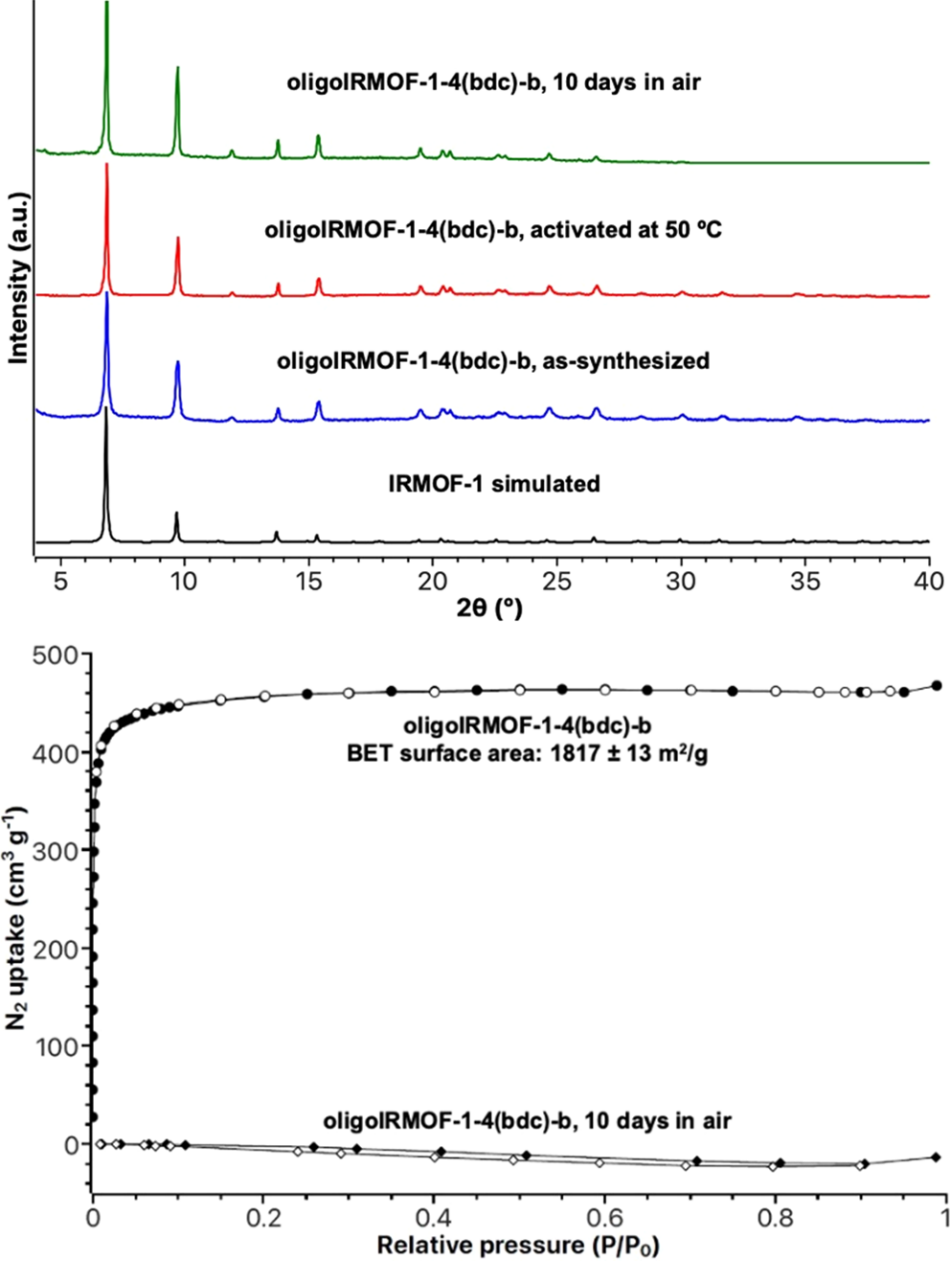
Top: PXRD of simulated IRMOF-1 (black), as-synthesized oligoIRMOF-1-4(bdc)-b (blue), oligoIRMOF-1-4(bdc)-b activated at 50 °C for 18 h (red), and oligoIRMOF-1–4(bdc)-b exposed to air for 10 days (green). Bottom: N_2_ isotherms of as-synthesized oligoIRMOF-1-4(bdc)-b and oligoIRMOF-1-4(bdc)-b exposed to air for 10 days. The filled and unfilled circles and diamonds correspond to adsorption and desorption, respectively.

To check stability, oligoIRMOF-1-4(bdc)-b was exposed to air for 10 days. As shown by powder X-ray diffraction (PXRD,Figure 1), crystallinity was retained, making the stability of oligoIRMOF-1-4(bdc)-b superior when compared to IRMOF-1 or previously reported oligoMOFs made from oligomers containing only two or three H_2_bdc units connected in a linear architecture^12^ According to a study of IRMOF-1 stability, IRMOF-1 begins to decompose upon exposure to ambient air for 10 min (evidenced by the presence of a new PXRD reflection at 2θ = 8.9°).^23^ By this standard, the oligoMOFs presented in this study display significantly improved stability, with the oligoMOF PXRD patterns remaining unchanged after 10 days of exposure to ambient air. However, the porosity of oligoIRMOF-1-4(bdc)-b was lost after exposure to air for 10 days. The retention of crystallinity but loss of surface area has been observed in other MOFs. Matzger and co-workers have shown that MOF collapse has been correlated with higher surface tension activation solvents due to capillary forces. Ultralow surface tension solvents such as *n*-hexane (17.9 mN/m) and perfluoropentane (9.4 mN/m) have been utilized as activation of fragile MOFs, giving similar performance to that of supercritical CO_2_ activation.^24,25^ Matzger and co-workers also studied Zn-HKUST-1 (Zn_3_(1,3,5-benzenetricarboxylate)_2_), which exhibited retention of crystallinity, but significant loss of porosity during activation.^26^ This phenomenon is related to pore collapse at the surface of the MOF crystal rather than the entire framework. The collapse of pores at the MOF crystal surface prevents even small molecules such as N_2_ from entering the bulk framework. Based on these studies, it is proposed that oligoIRMOF-1-4(bdc)-b undergoes a similar pore collapse at the crystal surface, leading to retention of bulk crystallinity but loss of accessible surface area (<20 m^2^/g). The collapse of surface pores of oligoIRMOF-1-4(bdc)-b is likely caused by moisture in ambient air, although more detailed studies are required to conclusively prove this hypothesis.

Utilizing a similar synthetic approach, an octameric ligand (**8(H**_**2**_**bdc)-b**) was synthesized (Scheme 1b). Due to the longer ligand length, oligoIRMOF synthesis with **8(H**_**2**_**bdc)-b** proved more challenging, initially producing only amorphous solids (Figure S6). Typically, the amines produced by thermal decomposition of DMF and DEF are sufficient to deprotonate the carboxylic acid groups of ligands used to produce MOFs.^27^ In the case of **8(H**_**2**_**bdc)-b**, the carboxylic acid groups may not easily deprotonate under standard MOF synthesis conditions due to intramolecular hydrogen bonding and increased charge density as the ligand becomes increasingly deprotonated. As such, it was found that the addition of an organic base was necessary to generate crystalline oligoIRMOFs with **8(H**_**2**_**bdc)-b**. Bases such as *N*,*N*-diisopropylethylamine (DIPEA) or triethylamine (TEA) allowed for the formation of the desired, crystalline oligoIRMOF-1-8(bdc)-b (Figure S7).

Optimization of conditions revealed that 8 equiv of DIPEA gave the best PXRD pattern, matching that of simulated IRMOF-1 (Figures 2 and S8). Activation of oligoIRMOF-1-8(bdc)-b was performed via solvent exchange with CH_2_Cl_2_ followed by heating under vacuum at 50 °C for 18 h. However, oligoIRMOF-1-8(bdc)-b did not register an accessible N_2_ BET surface area (Figure 2). Different activation methods, such as supercritical CO_2_ exchange and solvent exchange with *n*-hexane and diethyl ether (Et_2_O), were performed in an attempt to generate an accessible surface area from oligoIRMOF-1-8(bdc)-b (Figure S10). All activation methods produced materials with similar PXRD patterns; however, different gas adsorption behaviors were observed. Like the CH_2_Cl_2_ exchanged sample, supercritical CO_2_ activated samples showed no porosity (Figure S10). However, solvent exchange with *n*-hexane or Et_2_O produced oligoIRMOF-1-8(bdc)-b samples with a type II isotherm and an improved BET surface area (265 ± 18 m^2^/g for *n*-hexane exchanged; 413 ± 16 m^2^/g for Et_2_O exchanged).

**Figure 2:**
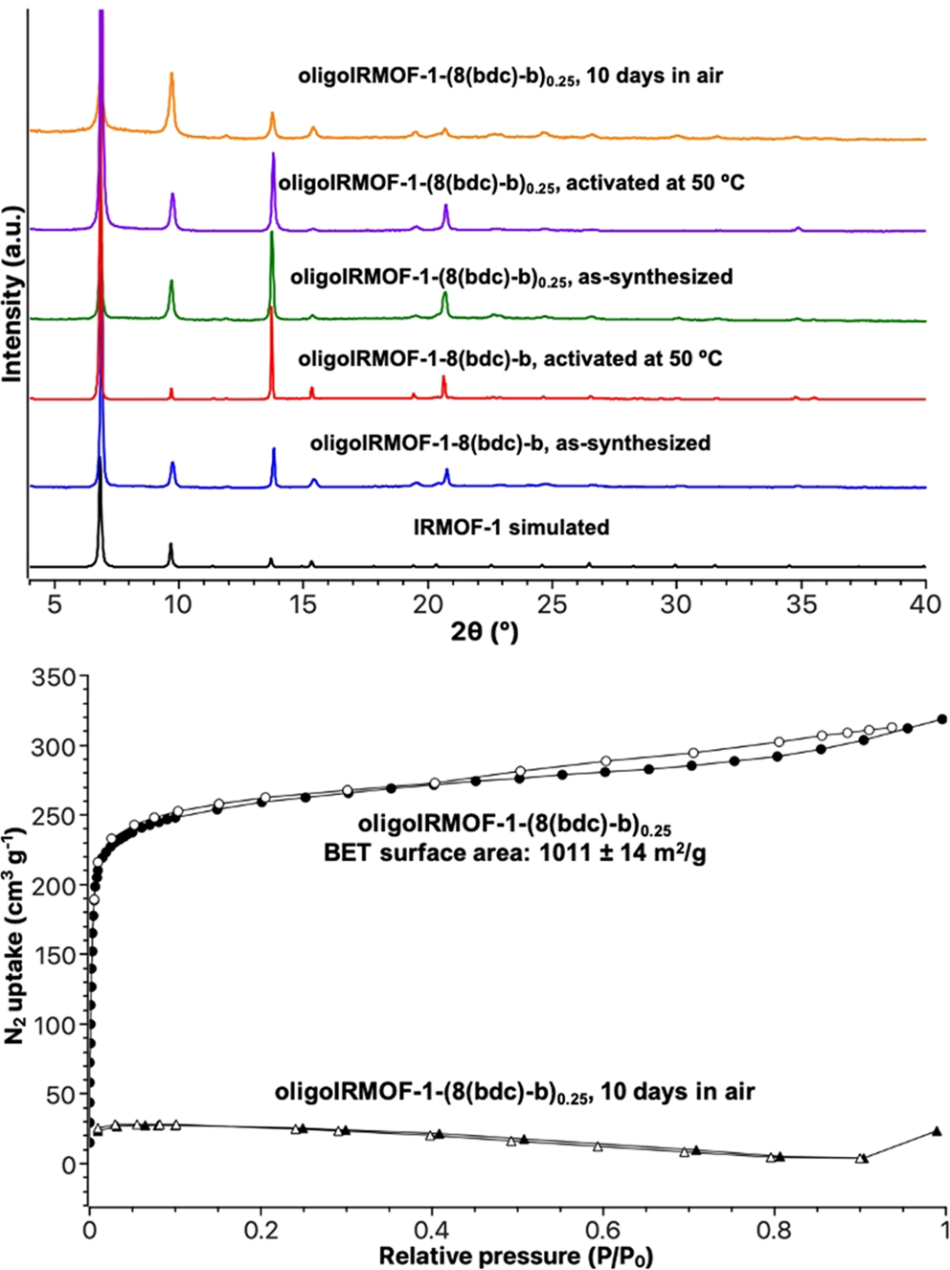
Top: PXRD of simulated IRMOF-1 (black), as-synthesized oligoIRMOF-1-8(bdc)-b (blue), oligoIRMOF-1-8(bdc)-b activated at 50 °C for 18 h (red), as-synthesized oligoIRMOF-1-(8(bdc)-b)_0.25_ (green), oligoIRMOF-1-(8(bdc)-b)_0.25_ activated at 50 °C for 18 h (purple), and oligoIRMOF-1-(8(bdc)-b)_0.25_ exposed to air for 10 days (orange). Bottom: N_2_ isotherms of as-synthesized oligoIRMOF-1-(8(bdc)b)_0.25_ and oligoIRMOF-1-(8(bdc)b)_0.25_ exposed to air for 10 days. The filled and unfilled circles and triangles correspond to adsorption and desorption, respectively.

To access greater porosity in these systems, **8(H**_**2**_**bdc)-b** and H_2_bdc were utilized in different ratios during MOF synthesis (Figure S11). A mixture containing 25% H_2_bdc and 75% **8(H**_**2**_**bdc)-b** combined with zinc nitrate at 100 °C gave an amorphous solid. Increasing the amount of H_2_bdc to 50% and using DMF as the solvent gave a material exhibiting low-intensity PXRD reflections indicative of an IRMOF. Changing the solvent to DEF improved the quality of the PXRD pattern but resulted in a material with no porosity (Figure S11). Further increasing the H_2_bdc percentage to 75% gave crystalline materials with either DMF or DEF as synthesis solvents (Figure 2). The activated MTV-MOF oligoIRMOF-1-(8(bdc)-b)_**0.25**_ was activated by soaking in CH_2_Cl_2_ followed by degassing at 50 °C for 18 h, giving a BET surface area of 1011 ± 14 m^2^/g. Although an enhanced surface area was obtained with this MTV-MOF, exposure to air for 10 days still resulted in a retention of crystallinity but loss of porosity (<75 m^2^/g). As described above, this is likely related to the pore collapse at the surface of the MOF.^24−26^

Finally, to compare the characteristics of branched and linear oligomer ligands, the linear linker (**4(H**_**2**_**bdc)-l**) with four H_2_bdc units was synthesized (Scheme 1c). OligoMOFs were synthesized from **4(H**_**2**_**bdc)-l** at 100 °C in DMF or DEF (Figure S12). These materials showed PXRD reflections corresponding to an IRMOF, but also reflections corresponding to zinc oxide (ZnO) at 2θ of 32, 35, and 36°. The linear architecture of **4(H**_**2**_**bdc)-l** seems to impact the synthesis conditions such that ZnO byproducts are formed during MOF synthesis, which are not observed when using the analogous branched ligand (e.g., **4(H**_**2**_**bdc)-b**). To avoid ZnO in the resulting oligoMOFs, various reaction conditions were explored. By lowering the reaction temperature to 80 °C and using DMF (instead of DEF) as a solvent, crystalline oligoIRMOF-1-4(bdc)-l was obtained (Figure 3). After solvent exchange with CH_2_Cl_2_ and activation at 50 °C for 18 h, this oligoMOF gave a BET value of 796 ± 25 m^2^/g, which is a slightly reduced value compared to IRMOF-1-4(bdc)-b, presumably due to the different geometry of **4(H**_**2**_**bdc)-l** (Figure 2). However, after exposure to air for 10 d, the PXRD of oligoIRMOF-1-4(bdc)-l showed maintenance of crystallinity, but again a loss of porosity (<20 m^2^/g).^24−26^ Interestingly, scanning electron microscopy (SEM) of all oligoMOF samples (Figures S27–S29) showed that oligoIRMOF-1-4(bdc)-l formed the smallest and most irregularly shaped crystallites.

**Figure 3:**
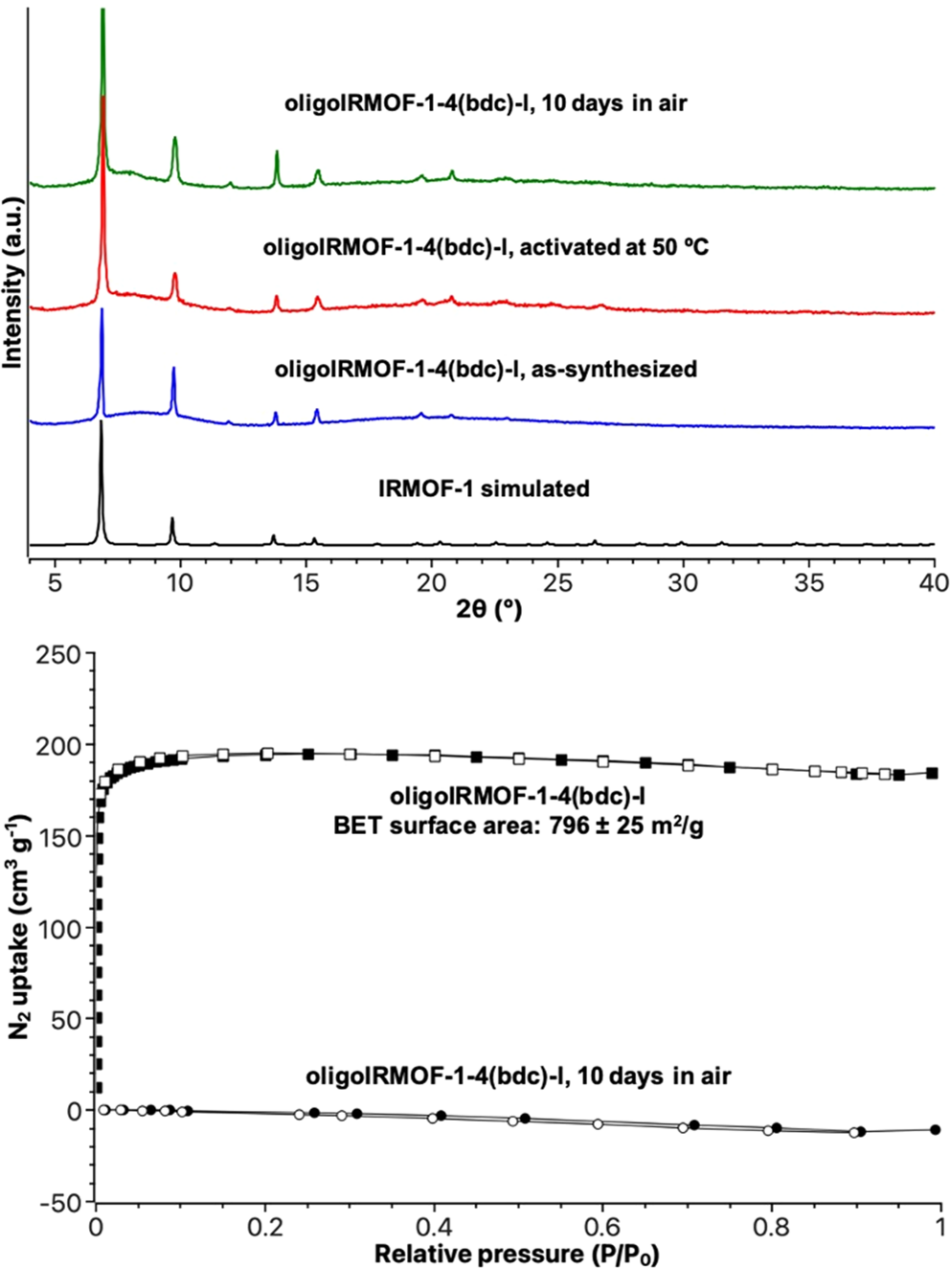
Top: PXRD of simulated IRMOF-1 (black), as-synthesized oligoIRMOF-1-4(bdc)-l (blue), oligoIRMOF-1-4(bdc)-l activated at 50 °C for 18 h (red), and oligoIRMOF-1-4(bdc)-l exposed to air for 10 days (green). Bottom: N_2_ isotherms of as-synthesized oligoIRMOF-1-4(bdc)-l and oligoIRMOF-1-4(bdc)-l exposed to air for 10 days. The filled and unfilled squares and circles correspond to adsorption and desorption, respectively.

The synthesis and characterization of a series of linear and branched oligoMOFs has been presented. It is quite remarkable that these highly branched, highly flexible oligomers can form rigid three-dimensional (3D) frameworks. Although the oligoMOFs have increased stability when compared to their molecular analogues, they largely seem to suffer from a loss of surface area due to pore collapse upon exposure to ambient conditions. Nevertheless, the chemistry of oligoMOFs is still in its infancy, and the versatile ligand architectures tolerated here bode well for future studies.

## Supporting Information Available

Materials, instrumentation, organic linker, MOF synthesis, and characterization details (PDF)
