# Supplementary material for: Metal–Organic Frameworks Constructed from Branched Oligomers
Source: Inorg Chem. 2024 Jan 12;63(4):1853–7. doi: 10.1021/acs.inorgchem.3c03452 (PMC10828985; doi:10.1021/acs.inorgchem.3c03452)
Supplement: Supplementary file 1 — ic3c03452_si_001.pdf [file ic3c03452_si_001.pdf]

SUPPORTING INFORMATION

Metal-Organic Frameworks Constructed from Branched Oligomers

Hyunyong Kim and Seth M. Cohen*

Department of Chemistry and Biochemistry, University of California, San Diego, La Jolla, California, 92093, United States

Email: scohen@ucsd.edu

General information. Starting materials were purchased from commercially available sources such as Sigma-Aldrich, Thermo Scientific, and Combi-blocks, Inc., and used without further purification. Dimethyl 2-hydroxyterephtalate (Tanabe, K. K.; Allen, C. A.; Cohen, S. M. *Angew. Chem. Int. Ed.* **2010**, *49*, 9730-9733.), diethyl 2-hydroxyterephtalate (Bolton, O.; Kim, J. *J. Mater. Chem.* **2007**, *17*, 1981-1988.), and dimethyl 2,5-dihydroxyterephtalate (Schneemann, A.; Vervoorts, P.; Hante, I.; Tu, M.; Wannapaiboon, S.; Sternemann, C.; Paulus, M.; Wieland, D. C. F.; Henke, S.; Fischer, R. A. *Chem. Mater*. **2018**, *30*, 1667-1676.) were synthesized by following reported procedures. Silica column chromatography was performed using a CombiFlash Rf+ automated system from TeledyneISCO (Lincoln, USA). ^{1}H and ^{13}C NMR spectra were collected by Bruker spectrometer operated at 300 MHz, or Jeol spectrometer operated at 400 MHz or 500 MHz. High resolution mass spectrometry (HRMS) and matrix-assisted laser desorption/ionization time-of-flight mass spectrometry (MALDI-TOF) were performed using an Agilent 6230 accurate-mass time-of-flight mass spectrometer and a Bruker Autoflex max MALDI-TOF mass spectrometer, respectively at the Molecular Mass Spectrometry Facility (MMSF) in the Department of Chemistry and Biochemistry at the University of California, San Diego.

Synthesis of oligomeric ligands 4(H_2bdc)-b, 8(H_2bdc)-b, and 4(H_2bdc)-l

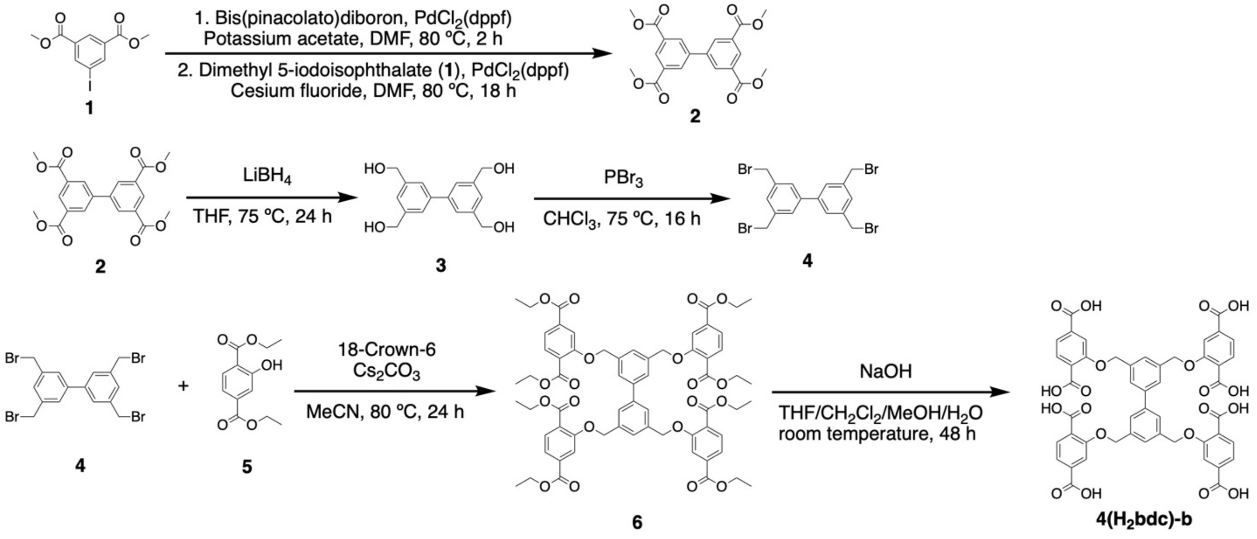

Figure S1. Synthesis of biphenyl with four bromomethyl groups (**4**) and branched tetramer (**4(H_2bdc)-b**).

Synthesis of 3,3’,5,5’-Tetrakis(methoxycarbonyl)biphenyl (2)

Dimethyl 5-iodoisophthalate (**1**) (2.0 g, 6.2 mmol) was dissolved in 40 mL of DMF. To the solution, bis(pinacolato)diboron (B_2Pin_2, 1.7 g, 6.9 mmol), and $PdCl_2$(dppf) (dppf = 1,1’-bis(diphenylphosphino)ferrocene) (150 mg, 188 μmol) were added. The reaction mixture was stirred at 80 ºC for 2 h. To the mixture, dimethyl 5-iodoisophthalate (**1**) (4.0 g, 12.5 mmol), $PdCl_2$(dppf) (150 mg, 188 μmol), and CsF (2.8 g, 18.8 mmol, dissolved in 10 mL of water) were added. The reaction mixture was heated at 80 ºC for 18 h with continuous stirring. After 18 h, the mixture was extracted with ethyl acetate, dried over $MgSO_4$, and dried under vacuum using a rotary evaporator. The crude material was purified by silica column chromatography (hexane/ethyl acetate, R_f: 0.38, Hex:EA=2:1) and dried under vacuum to give a beige solid. Yield: 1.9 g (78%). ^{1}H NMR (400 MHz, $CDCl_3$): δ 8.73 (t, J = 1.6 Hz, 2H), 8.52 (d, J = 1.6 Hz, 4H), 4.00 (s, 12H); ^{13}C NMR (100 MHz, $CDCl_3$): δ 166.13, 140.02, 132.47, 131.68, 130.39, 52.76; HRMS m/z calculated for $[C_{20}H_{18}O_8+H]^+$: 387.1074, found: 387.1076.

Synthesis of [1,1'-biphenyl]-3,3',5,5'-tetrayltetramethanol (3)

3,3’,5,5’-Tetrakis(methoxycarbonyl)biphenyl (**2**) (1.9 g, 4.9 mmol) was dispersed in 20 mL of THF. To the mixture, 2 M $LiBH_4$ in THF (73 mL, 147 mmol) was slowly added. The reaction mixture was stirred at 75 ºC for 24 h. The volatile solvent was removed under vacuum using a rotary evaporator. To the mixture, 60 mL of water was slowly added. The resulting white precipitate was collected by filtration, washed with water, and dried under vacuum to give a white solid. Yield: 1.3 g (66%). ^{1}H NMR (400 MHz, DMSO-d_6): δ 7.47 (s, 4H), 7.25 (s, 2H), 5.26 (t, J = 5.7 Hz, 4H), 4.56 (d, J = 5.6 Hz, 8H); ^{13}C NMR (125 MHz, DMSO-d_6): δ 143.01, 140.02, 123.57, 123.00, 62.96; HRMS m/z calculated for $[C_{16}H_{18}O_4-H]^-$: 273.1132, found: 273.1136.

Synthesis of 3,3',5,5'-tetrakis(bromomethyl)-1,1'-biphenyl (4)

[1,1'-Biphenyl]-3,3',5,5'-tetrayltetramethanol (**3**) (890 mg, 3.2 mmol) was dispersed into 135 mL of chloroform. Phosphorous tribromide (15 mL, 162 mmol) was slowly added. The reaction

mixture was stirred at 75 ºC for 16 h. Volatile solvents were removed under vacuum using a rotary evaporator. Water was slowly added until the bubble ceased. The resulting light beige solid was collected by filtration, washed with water, and dried under vacuum to give a light beige powder. Yield: 1.7 g (98%). ^{1}H NMR (300 MHz, $CDCl_3$): δ 7.52 (d, J = 1.7 Hz, 4H), 7.44 (t, J = 1.7 Hz, 2H), 4.54 (s, 8H); ^{13}C NMR (100 MHz, $CDCl_3$): δ 141.38, 139.32, 129.12, 128.03, 32.72; MS (EI) *m/z* calculated for $[C_{16}H_{14}Br_4]^+$: 521.78, found: 521.77.

Synthesis of octaethyl 2,2',2'',2'''-(([1,1'-biphenyl]-3,3',5,5'-tetrayltetrakis(methylene))tetrakis(oxy))tetraterephthalate (6)

3,3',5,5'-Tetrakis(bromomethyl)-1,1'-biphenyl (**4**) (200 mg, 380 μmol), diethyl 2-hydroxyterephthalate (**5**) (900 mg, 3.8 mmol), 18-crown-6 (80 mg, 304 μmol), and cesium carbonate (1.2 g, 3.8 mmol) were dispersed into 30 mL of acetonitrile. The reaction mixture was stirred at 80 ºC for 24 h. The solid was removed by filtration, and washed with CH_2Cl_2. The filtrate was dried under vacuum using a rotary evaporator, triturated with MeOH, washed with EtOH and acetone, and dried under vacuum to give a white solid. Yield: 325 mg (74%). ^{1}H NMR (500 MHz, $CDCl_3$): δ 7.87 (d, J = 1.6 Hz, 4H), 7.84 (d, J = 8.0 Hz, 4H), 7.75 (d, J = 1.4 Hz, 4H), 7.68 (dd, J = 8.0, 1.4 Hz, 4H), 7.65 (s, 2H), 5.31 (s, 8H), 4.39 (q, J = 7.1 Hz, 8H), 4.30 (q, J = 7.1 Hz, 8H), 1.39 (t, J = 7.1 Hz, 12H), 1.22 (t, J = 7.1 Hz, 12H); ^{13}C NMR (100 MHz, $CDCl_3$): δ 165.95, 165.80, 157.76, 141.26, 137.46, 134.76, 131.66, 125.96, 125.35, 125.25, 121.81, 114.44, 70.87, 61.66, 61.41, 14.41, 14.25; HRMS *m/z* calculated for $[C_{64}H_{66}O_{20}+NH_4]^+$: 1172.4486, found: 1172.4490; calculated for $[C_{64}H_{66}O_{20}+Na]^+$: 1170.4040, found: 1170.4042.

Synthesis of 4(H_2bdc)-b

Compound **6** (300 mg, 260 μmol) was dissolved in 10 mL of THF, 10 mL of water, 15 mL of MeOH, and 20 mL of CH_2Cl_2. To the solution, NaOH (1.6 g, 39 mmol) was added. The reaction mixture was stirred at room temperature for 48 h. The volatile solvent was removed under vacuum using a rotary evaporator, and acidified with 1 M HCl until the pH reached 1. The resulting precipitate was collected by filtration, washed with water, and dried under vacuum to

obtain a white solid. Yield: 230 mg (95%). ^{1}H NMR (500 MHz, DMSO-d_6): δ 7.86 (s, 4H), 7.79-7.70 (m, 10H), 7.60 (dd, J = 7.9, 1.3 Hz, 4H), 5.35 (s, 2H); ^{13}C NMR (100 MHz, DMSO-d_6): δ 167.22, 166.67, 156.74, 140.22, 137.81, 134.60, 130.66, 126.25, 125.67, 125.44, 121.51, 114.08, 69.99; HRMS m/z calculated for $[C_{48}H_{34}O_{20}\text{-}H]^-$: 929.1571, found: 929.1583.

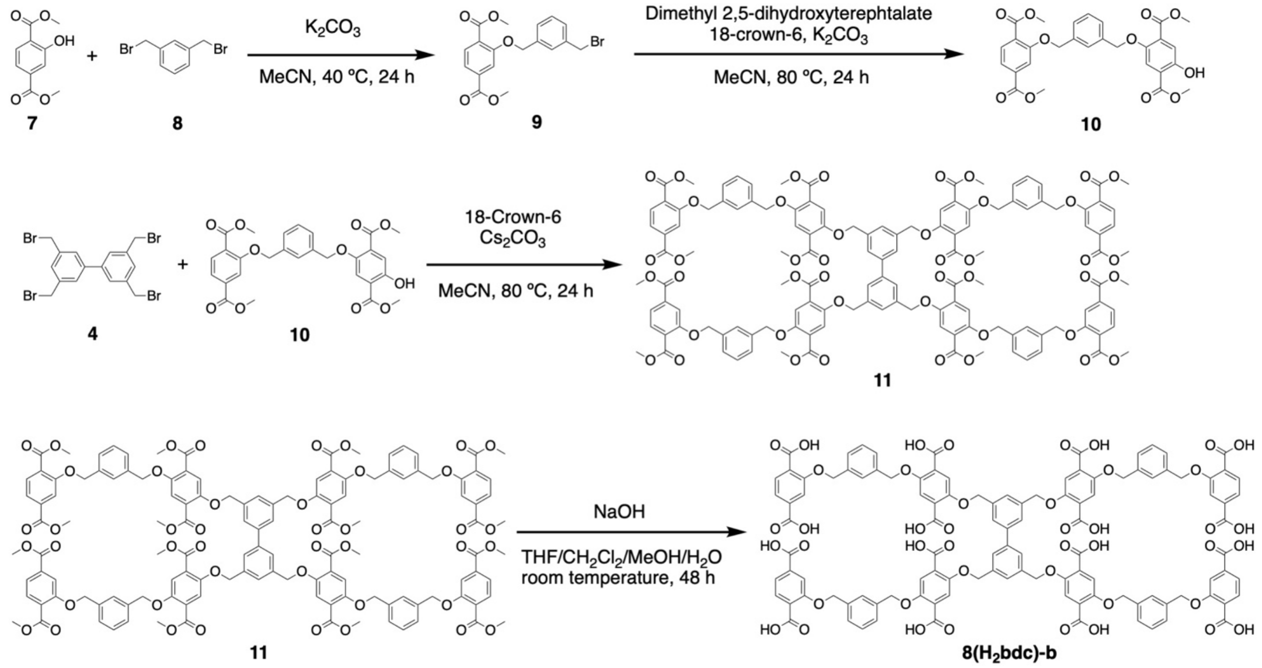

Figure S2. Synthesis of dimethyl 2-((3-(bromomethyl)benzyl)oxy)terephthalate (**9**), dimethyl 2-((3-((2,5-bis(methoxycarbonyl)phenoxy)methyl)benzyl)oxy)-5-hydroxyterephthalate (**10**), and octamer (**8(H_2bdc)-b**).

Synthesis of dimethyl 2-((3-(bromomethyl)benzyl)oxy)terephthalate (9)

Dimethyl 2-hydroxyterephthlate (**7**) (2.0 g, 9.5 mmol) was dissolved in 95 mL of acetonitrile. To the solution, 1,3-bis(bromomethyl)benzene (**8**) (6.3 g, 23.8 mmol) and potassium carbonate (2.0 g, 14.3 mmol) were added. The reaction mixture was stirred at 40 ºC for 24 h. The remaining solid was removed by filtration and the filtrate was dried under vacuum using a rotary evaporator. The crude compound was isolated by silica column chromatography (hexane/ethyl acetate, R_f: 0.57, Hex:EA=2:1) and dried under vacuum to give a white solid. Yield: 2.4 g (64%). ^{1}H

NMR (400 MHz, $CDCl_3$): δ 7.86 (d, J = 7.9 Hz, 1H), 7.72-7.64 (m, 2H), 7.56 (s, 1H), 7.46-7.43 (m, 1H), 7.40-7.34 (m, 1H), 5.22 (s, 2H), 4.52 (s, 2H), 3.94 (d, J = 0.8 Hz, 6H); ^{13}C NMR (100 MHz, $CDCl_3$): δ 166.40, 166.26, 157.72, 138.28, 137.15, 134.55, 131.84, 129.25, 128.76, 127.70, 127.09, 124.94, 121.87, 114.50, 70.44, 52.70, 52.55, 33.48; HRMS *m/z* calculated for $[C_{18}H_{17}BrO_5+Na]^+$: 415.0152, found: 415.0155.

Synthesis of dimethyl 2-((3-((2,5-bis(methoxycarbonyl)phenoxy)methyl)benzyl)oxy)-5-hydroxyterephthalate (10)

Dimethyl 2-((3-(bromomethyl)benzyl)oxy)terephthalate (**9**) (1.5 g, 3.8 mmol) was dissolved in 120 mL of acetonitrile. To the solution, dimethyl 2,5-dihydroxyterephthalate (2.2 g, 9.5 mmol), 18-crown-6 (200 mg, 763 μmol), and potassium carbonate (1.3 g, 9.5 mmol) were added. The reaction mixture was stirred at 80 ºC for 24 h. The remaining solid was removed by filtration. The crude compound was isolated by silica column chromatography (hexane/ethyl acetate, R_f: 0.24, Hex:EA=2:1) and dried under vacuum to give a white solid. Yield: 1.1 g (54%). ^{1}H NMR (400 MHz, $CDCl_3$): δ 10.32 (s, 1H), 7.85 (d, J = 8.0 Hz, 1H), 7.72-7.63 (m, 2H), 7.57 (s, 1H), 7.52-7.39 (m, 4H), 7.37 (s, 1H), 5.25 (s, 2H), 5.13 (s, 2H), 3.97 (s, 3H), 3.93 (s, 3H), 3.91 (s, 3H), 3.89 (s, 3H); ^{13}C NMR (100 MHz, $CDCl_3$): δ 169.65, 166.34, 166.27, 165.82, 157.83, 155.59, 149.79, 137.17, 136.76, 134.52, 131.75, 129.07, 129.00, 126.87, 126.67, 125.69, 121.78, 120.42, 115.03, 114.93, 114.61, 71.97, 70.78, 52.84, 52.68, 52.61, 52.46; HRMS *m/z* calculated for $[C_{28}H_{26}O_{11}+NH_4]^+$: 556.1813, found: 556.1824; calculated for $[C_{28}H_{26}O_{11}+Na]^+$: 561.1367, found: 561.1372.

Synthesis of octamethyl 5,5',5'',5'''-(([1,1'-biphenyl]-3,3',5,5'-tetrayltetrakis(methylene))tetrakis(oxy))tetrakis(2-((3-((2,5-bis(methoxycarbonyl)phenoxy)methyl)benzyl)oxy)terephthalate) (11)

3,3',5,5'-Tetrakis(bromomethyl)-1,1'-biphenyl (**4**) (80 mg, 152 μmol), dimethyl 2-((3-((2,5-bis(methoxycarbonyl)phenoxy)methyl)benzyl)oxy)-5-hydroxyterephthalate (**10**) (819 mg, 1.52 mmol), 18-crown-6 (32 mg, 122 μmol), and cesium carbonate (500 mg, 1.52 mmol) were dissolved in 20 mL of acetonitrile. The reaction mixture was stirred at 80 ºC for 24 h. The solid was

removed by filtration, and washed with CH_2Cl_2. The filtrate was dried under vacuum using a rotary evaporator, triturated with MeOH, washed with EtOH and acetone, and dried under vacuum to give a white solid. Yield: 280 mg (78%). ^{1}H NMR (400 MHz, $CDCl_3$): δ 7.85 (d, J = 8.0 Hz, 5H), 7.75 (s, 4H), 7.72-7.64 (m, 9H), 7.59 (s, 5H), 7.54-7.40 (m, 19H), 5.76 (s, 4H), 5.25 (s, 10H), 5.18 (s, 10H), 3.93 (s, 12H), 3.90 (d, J = 3.5 Hz, 24H), 3.86 (s, 12H); ^{13}C NMR (100 MHz, $CDCl_3$): δ 166.33, 166.27, 165.75, 165.68, 157.83, 153.09, 149.70, 136.88, 136.79, 134.52, 131.76, 129.10, 126.79, 126.73, 126.06, 125.60, 125.15, 124.93, 121.78, 121.17, 117.07, 114.58, 93.97, 71.54, 70.76, 52.68, 52.57, 52.48; MS (MALDI-TOF, matrix: α-cyano-4-hydroxycinnamic acid) m/z calculated for $[C_{128}H_{114}O_{44}+Na]^+$: 2377.66, found: 2377.89; calculated for $[C_{128}H_{114}O_{44}+K]^+$: 2393.63, found: 2393.86.

Synthesis of 8(H_2bdc)-b

Compound **11** (250 mg, 106 μmol) was dissolved in 10 mL of THF, 10 mL of water, 20 mL of MeOH, and 20 mL of CH_2Cl_2. To the solution, NaOH (1.3 g, 32 mmol) was added. The reaction mixture was stirred at room temperature for 48 h. The volatile solvent was removed under vacuum by a rotary evaporator, and acidified with 1 M HCl until the pH reached 1. The resulting precipitate was collected by filtration, washed with water, and dried under vacuum to obtain a white solid. Yield: 210 mg (93%). ^{1}H NMR (400 MHz, DMSO-d_6): δ 7.71-7.63 (m, 9H), 7.61-7.50 (m, 14H), 7.48-7.34 (m, 19H), 5.72 (s, 4H), 5.23 (s, 10H), 5.15 (s, 10H); ^{13}C NMR (100 MHz, DMSO-d_6): δ 167.15, 166.66, 166.52, 166.33, 156.63, 151.81, 148.55, 137.08, 136.92, 134.53, 130.52, 128.58, 126.76, 126.71, 126.60, 126.27, 126.08, 125.74, 121.44, 119.71, 115.81, 114.01, 93.90, 70.51, 69.81; MS (MALDI-TOF, matrix: α-Cyano-4-hydroxycinnamic acid) m/z calculated for $[C_{112}H_{82}O_{44}+Na]^+$: 2153.41, found: 2153.49; calculated for $[C_{112}H_{82}O_{44}+2Na-H]^+$: 2175.39, found: 2175.47.

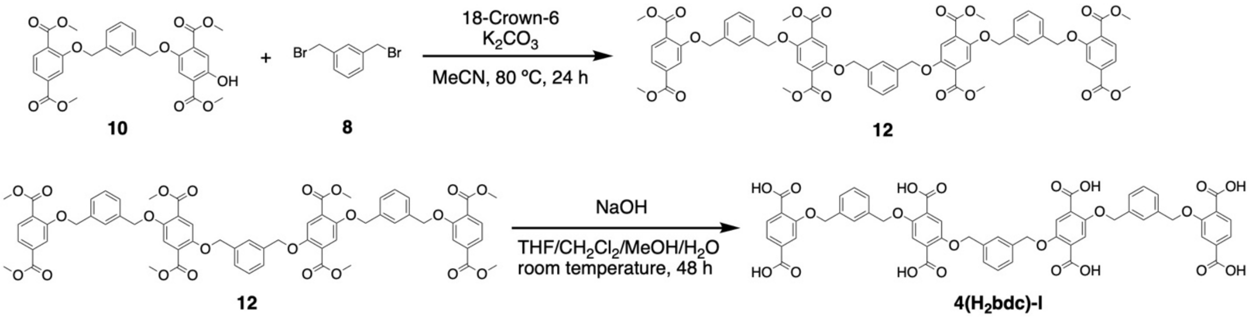

Figure S3. Synthesis of linear tetramer (**4(H_2bdc)-l**).

Synthesis of tetramethyl 5,5'-((1,3-phenylenebis(methylene))bis(oxy))bis(2-((3-((2,5-bis(methoxycarbonyl)phenoxy)methyl)benzyl)oxy)terephthalate) (12)

Dimethyl 2-((3-((2,5-bis(methoxycarbonyl)phenoxy)methyl)benzyl)oxy)-5-hydroxyterephthalate (**10**) (490 mg, 917 µmol), and 1,3-bis(bromomethyl)benzene (**8**) (110 mg, 417 µmol) were dissolved in 20 mL of acetonitrile. To the solution, 18-crown-6 (88 mg, 333 µmol), and potassium carbonate (580 mg, 4.17 mmol) were added. The reaction mixture was stirred at 80 ºC for 24 h. The remaining solid was removed by filtration, washed with CH_2Cl_2, and dried under vacuum by using a rotary evaporator. The crude material was triturated by MeOH, washed with EtOH and acetone, and dried under vacuum to obtain a white solid. Yield: 310 mg (63%). ^{1}H NMR (400 MHz, $CDCl_3$): δ 7.85 (d, J = 8.0 Hz, 2H), 7.73-7.63 (m, 4H), 7.59(s, 3H), 7.54-7.40 (m, 13H), 5.25 (s, 4H), 5.17 (s, 8H), 3.96-3.88 (m, 24H); ^{13}C NMR (100 MHz, $CDCl_3$): δ 166.35, 166.27, 166.00, 157.83, 151.91, 137.07, 137.04, 136.77, 134.53, 131.77, 129.07, 126.82, 126.69, 125.78, 125.66, 125.01, 121.79, 117.89, 114.59, 71.77, 70.78, 52.67, 52.60, 52.59, 52.48; HRMS *m/z* calculated for $[C_{64}H_{58}O_{22}+NH_4]^+$: 1196.3758, found: 1196.3764; calculated for $[C_{64}H_{58}O_{22}+Na]^+$: 1201.3312, found: 1201.3305.

Synthesis of 4(H_2bdc)-l

Compound **12** (310 mg, 263 µmol) was dissolved in 10 mL of THF, 10 mL of water, 15 mL of MeOH, and 20 mL of CH_2Cl_2. To the solution, NaOH (1.6 g, 39 mmol) was added. The reaction mixture was stirred at room temperature for 48 h. The volatile solvent was removed

under vacuum using rotary evaporator, acidified with 1 M HCl until the pH reached 1. The resulting precipitate was collected by filtration, washed with water, and dried under vacuum to obtain a white solid. Yield: 260 mg (93%). ^{1}H NMR (400 MHz, DMSO-d_6): δ 7.75-7.66 (m, 4H), 7.59 (dt, J = 9.2, 8.0 Hz, 5H), 7.52-7.38 (m, 13H), 5.27 (s, 4H), 5.17 (s, 8H); ^{13}C NMR (100 MHz, DMSO-d_6): δ 167.16, 166.83, 166.65, 156.62, 150.49, 137.20, 137.15, 136.90, 134.51, 130.50, 128.56, 126.71, 126.57, 126.29, 126.09, 125.76, 121.43, 116.19, 114.00, 70.65, 69.80; HRMS m/z calculated for $[C_{56}H_{42}O_{22}\text{-}H]^-$: 1065.2095, found: 1065.2086.

Synthesis of oligoIRMOF-1-4(bdc)-b. $Zn(NO_3)_2{\cdot}6H_2O$ (155 mg, 0.52 mmol) and **4(H$_2$bdc)-b** (30 mg, 32 μmol) were dissolved in 4 mL of DEF in 20 mL scintillation vial. The vial was placed in an oven. The temperature was raised from room temperature to 100 °C at 0.5 °C/min, held for 72 h. The vial was removed and allowed to cool to room temperature. The mother liquor was decanted and the oligoMOF was washed with fresh DMF three times and rinsed with CH_2Cl_2 three times. Prior to analysis, oligoMOF samples were activated under vacuum at 50 °C for 18 h. Yield: 24 mg (77%, molecular formula: Zn_4O(**4(bdc)-b**)$_{0.75}$).

Synthesis of oligoIRMOF-1-8(bdc)-b in the presence of DIPEA. $Zn(NO_3)_2{\cdot}6H_2O$ (335 mg, 1.13 mmol) and **8(H$_2$bdc)-b** (80 mg, 38 μmol) were dissolved in 5 mL of DEF in 20 mL scintillation vial. To the solution, DIPEA (52 μL, 300 μmol) was added. The vial was placed in a preheated oven at 80 °C for 72 h. The vial was removed and allowed to cool to room temperature. The mother liquor was decanted and MOF sample was washed with fresh DMF three times and rinsed with CH_2Cl_2 three times. Prior to analysis, oligoMOF samples were activated under vacuum at 50 °C for 18 h. Yield: 35 mg (87%, molecular formula: Zn_4O(**8(bdc)-b**)$_{0.375}$).

Alternative activation method for oligoIRMOF-1-8(bdc)-b

Supercritical CO_2 drying. The process was performed by a Tousimis Samdri PVT-3D critical point drier. Liquid CO_2 was used to exchange CH_2Cl_2 three times. The material was heated above 31 °C (P = 1072 psi), the critical point of CO_2. The gas state CO_2 slowly released at 0.1-

1 cm^3/min. The sample was activated at 25 °C for 18 h before nitrogen adsorption experiment.

Solvent exchange with *n*-hexane or diethyl ether. As-synthesized oligoMOFs were washed with DMF three times (3×20 mL) and exchanged with CH_2Cl_2 three times (3×20 mL). The CH_2Cl_2 exchanged samples were immersed in 20 mL of *n*-hexane or diethyl ether for solvent exchange and repeated three times. The samples were evacuated under vacuum at 25 °C for 18 h before nitrogen adsorption experiment.

Synthesis of oligoIRMOF-1-8(bdc)-b in the presence of H_2bdc (oligoIRMOF-1-(8(bdc)-b)$_{0.25}$). $Zn(NO_3)_2·6H_2O$ (89 mg, 0.30 mmol), **8(H_2bdc)-b** (20 mg, 9.4 µmol), and terephthalic acid (4.7 mg, 28 µmol) were dissolved in 1.2 mL of DEF in 1.5 dram vial. The vial was placed in a preheated oven at 100 °C for 72 h. The vial was removed and allowed to cool to room temperature. The mother liquor was decanted and oligoMOF sample was washed with fresh DMF three times and rinsed with CH_2Cl_2 three times. Prior to analysis, oligoMOF samples were activated under vacuum at 50 °C for 18 h. Yield: 19 mg (63%, molecular formula: Zn_4O(bdc)$_{2.25}$(**8(bdc)-b**)$_{0.094}$).

Synthesis of oligoIRMOF-1-4(bdc)-l. $Zn(NO_3)_2·6H_2O$ (268 mg, 0.90 mmol) and **4(H_2bdc)-l** (60 mg, 56 µmol) were dissolved in 7 mL of DMF in 20 mL scintillation vial. The vial was placed in a preheated oven at 80 °C for 72 h. The vial was removed and allowed to cool to room temperature. The mother liquor was decanted and oligoMOF sample was washed with fresh DMF three times and rinsed with CH_2Cl_2 three times. Prior to analysis, oligoMOF samples were activated under vacuum at 50 °C for 18 h. Yield: 44 mg (73%, molecular formula: Zn_4O(**4(bdc)-l**)$_{0.75}$).

Nitrogen adsorption isotherm analysis

Around 20-50 mg of dried oligoMOF was transferred to a preweighed sample tube and degassed at 50 °C on a Micromeritics ASAP 2020 Adsorption analyzer for 18 h and the sample tube was

then reweighed to determine the sample mass. Nitrogen adsorption was then performed at 77 K on the same instrument. BET surface areas were determined from sorption isotherms using the BETSI program.

Powder X-Ray Diffraction (PXRD)

Dry oligoMOF powder (10 mg) was mounted on a silicon sample holder. PXRD data were collected at ambient temperature on a Bruker D8 Advance diffractometer using LynxEye detector at 40 kV, 40 mA for Cu Kα (λ = 1.5418 Å), with a scan speed of 0.5 sec/step, a step size of 0.02° in 2θ, and a 2θ range of 4-40°.

PXRD patterns and N_2 isotherms of oligoIRMOF-1-4(bdc)-b under different conditions

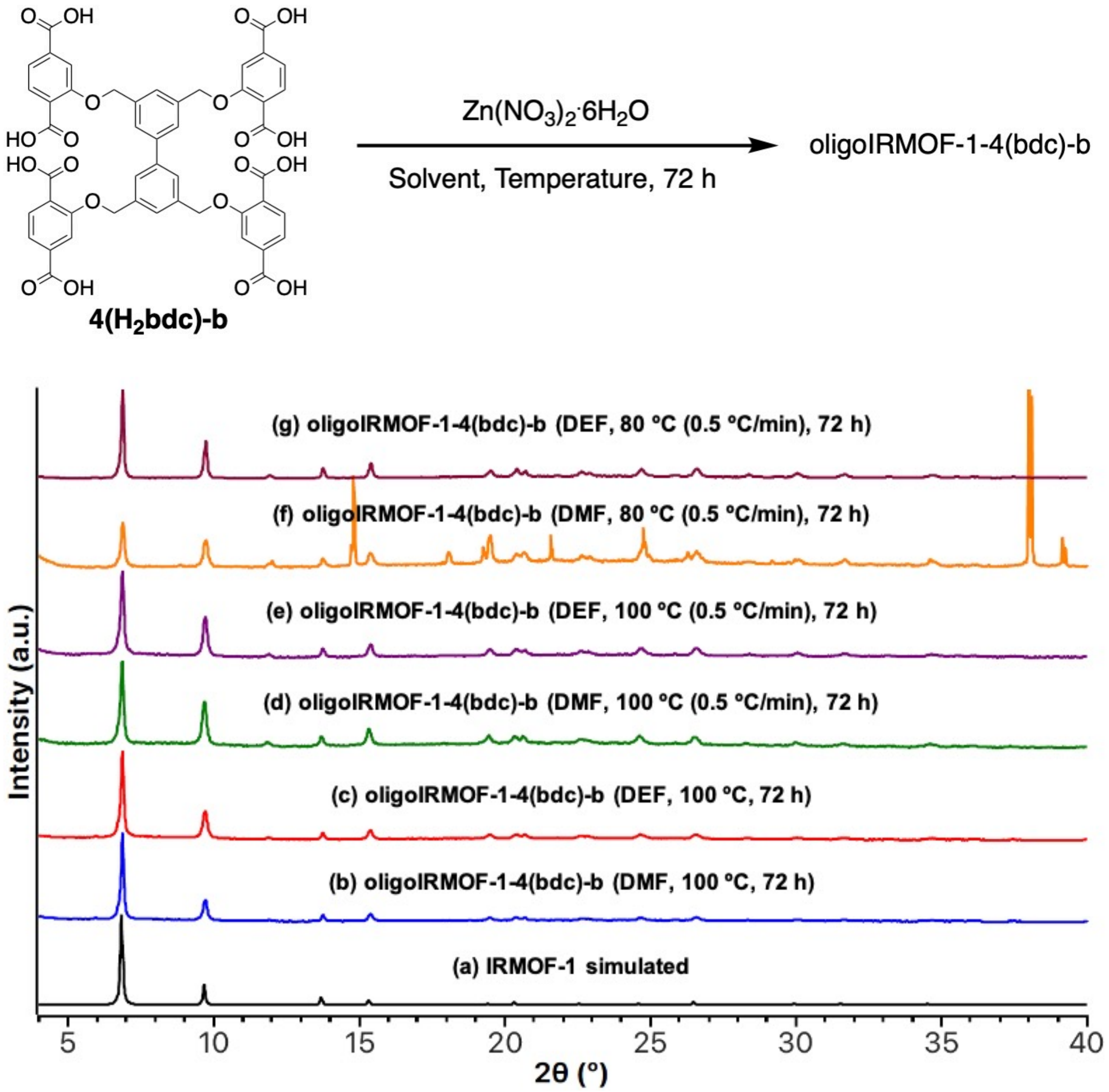

Figure S4. *Top*: Synthetic scheme for oligoIRMOF-1-4(bdc)-b. *Bottom:* PXRD patterns of (a) simulated IRMOF-1, oligoIRMOF-1-4(bdc)-b prepared under various conditions: (b) DMF, 100 ºC, 72 h, (c) DEF, 100 ºC, 72 h, (d) DMF, 100 ºC (slow heating: 0.5 ºC/min), 72 h, (e) DEF, 100 ºC (slow heating: 0.5 ºC/min), 72 h, (f) DMF, 80 ºC (slow heating: 0.5 ºC/min), 72 h, and (g) DEF, 80 ºC (slow heating: 0.5 ºC/min), 72 h.

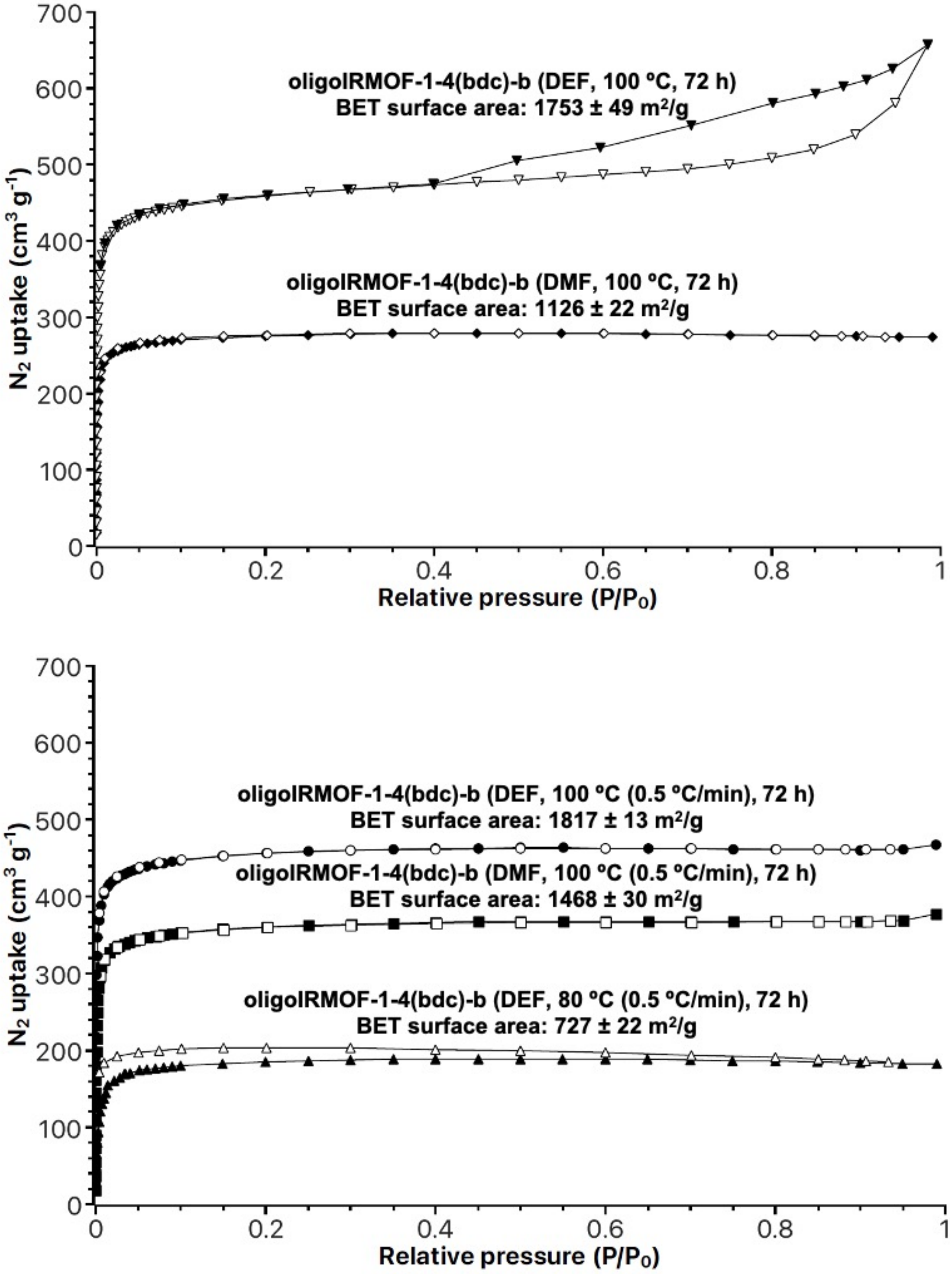

Figure S5. *Top*: N_2 adsorption isotherms of oligoIRMOF-1-4(bdc)-b synthesized under isothermal conditions. *Bottom*: N_2 adsorption isotherms of oligoIRMOF-1-4(bdc)-b synthesized using slow, ramped heating (0.5 ºC/min). The filled and unfilled symbols are corresponding to adsorption and desorption, respectively.

PXRD patterns and N_2 isotherms of oligoIRMOF-1-8(bdc)-b under different conditions

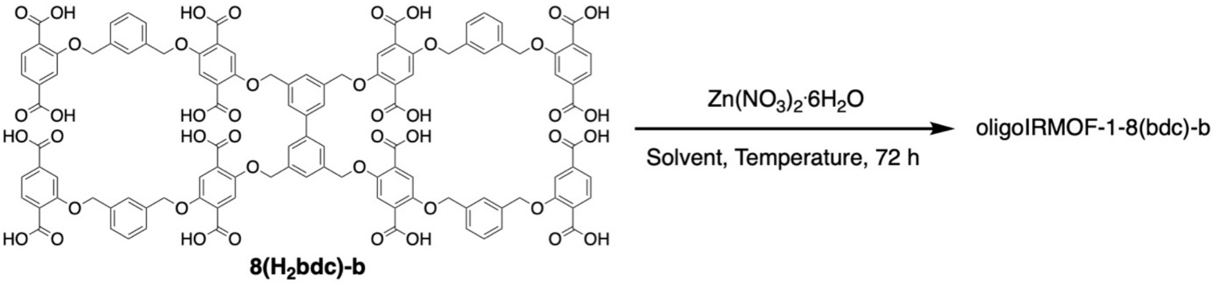

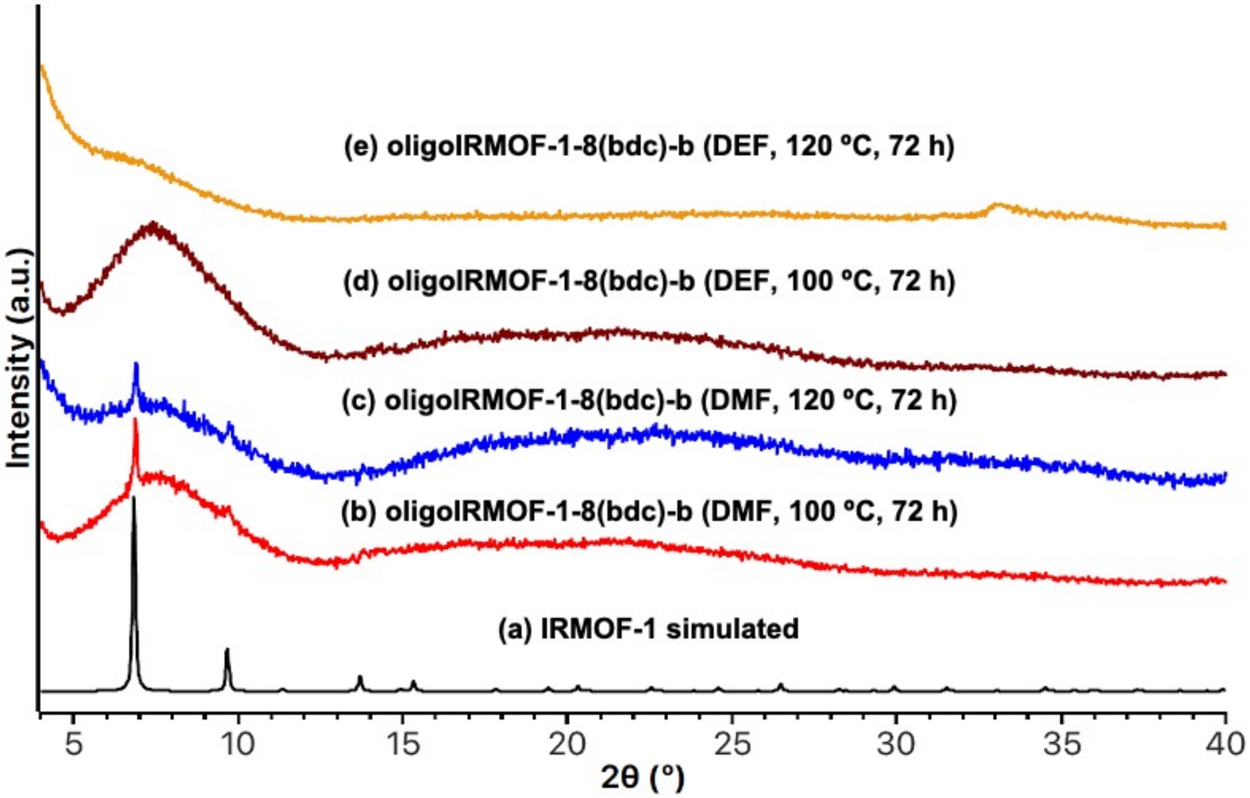

Figure S6. *Top*: Synthetic scheme for oligoIRMOF-1-8(bdc)-b without the use of an external base. *Bottom*: PXRD patterns of (a) simulated IRMOF-1, oligoIRMOF-1-8(bdc)-b prepared under various conditions: (b) DMF, 100 ºC, 72 h, (c) DMF, 120 ºC, 72 h, (d) DEF, 100 ºC, 72 h, and (e) DEF, 120 ºC, 72 h.

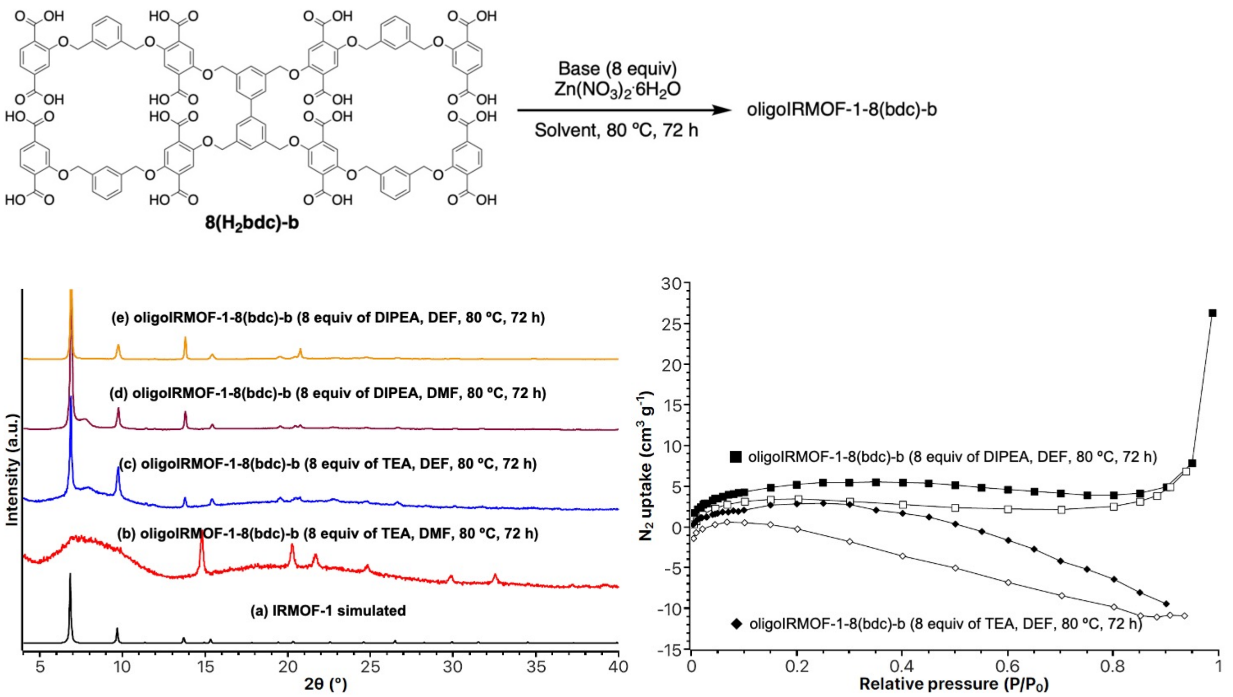

Figure S7. *Top*: Synthetic scheme for oligoIRMOF-1-8(bdc)-b in the presence of bases, such as DIPEA and TEA. *Bottom Left*: PXRD patterns of (a) simulated IRMOF-1, oligoIRMOF-1-8(bdc)-b prepared under different conditions: (b) TEA (8 equiv), DMF, 80 ºC, 72 h, (c) TEA (8 equiv), DEF, 80 ºC, 72 h, (d) DIPEA (8 equiv), DMF, 80 ºC, 72 h, and (e) DIPEA (8 equiv), DEF, 80 ºC, 72 h. *Bottom Right*: N_2 adsorption isotherms of oligoIRMOF-1-8(bdc)-b in the presence of DIPEA or TEA as a base. The filled and unfilled symbols are corresponding to adsorption and desorption, respectively.

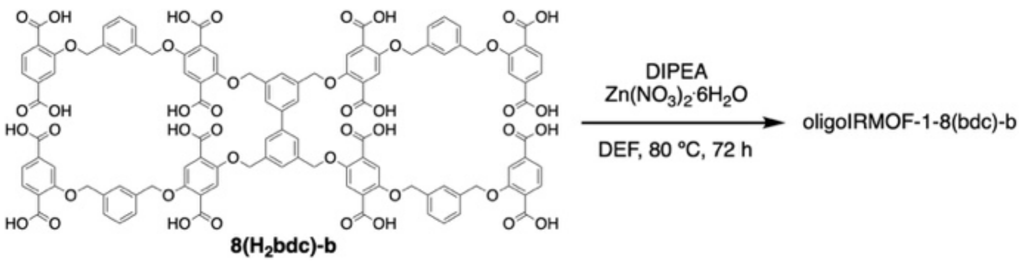

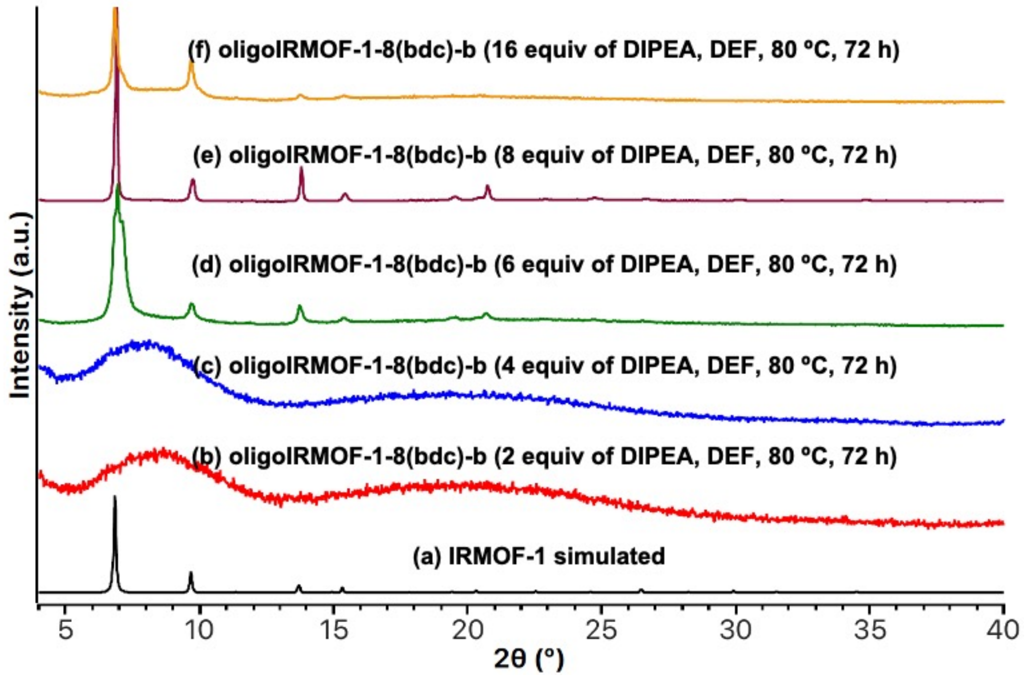

Figure S8. *Top*: S Synthetic scheme for oligoIRMOF-1-8(bdc)-b in the presence of different amounts of DIPEA. *Bottom*: PXRD patterns of (a) simulated IRMOF-1, (b) oligoIRMOF-1-8(bdc)-b prepared in the presence of 2 equiv of DIPEA, (c) 4 equiv of DIPEA, (d) 6 equiv of DIPEA, (e) 8 equiv of DIPEA, and (f) 16 equiv of DIPEA.

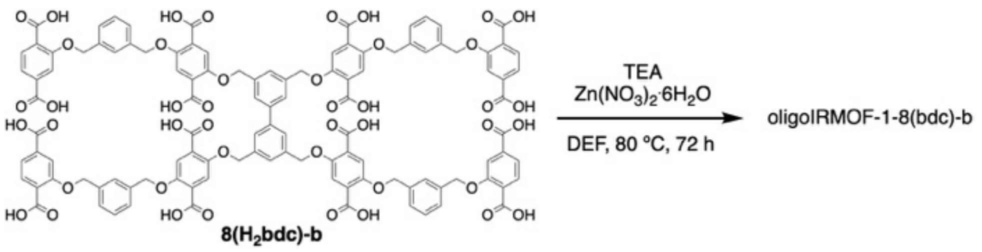

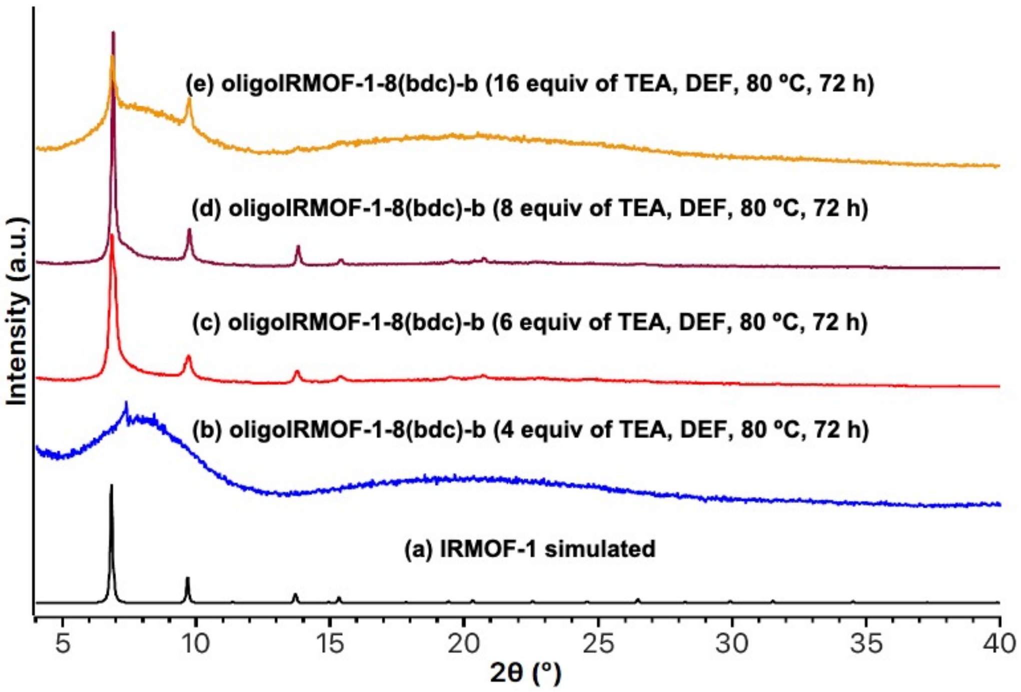

Figure S9. *Top*: Synthetic scheme for oligoIRMOF-1-8(bdc)-b in the presence of different amounts of TEA. *Bottom*: PXRD patterns of (a) simulated IRMOF-1, (b) oligoIRMOF-1-8(bdc)-b prepared in the presence of 4 equiv of TEA, (c) 6 equiv of TEA, (d) 8 equiv of TEA, and (e) 16 equiv of TEA.

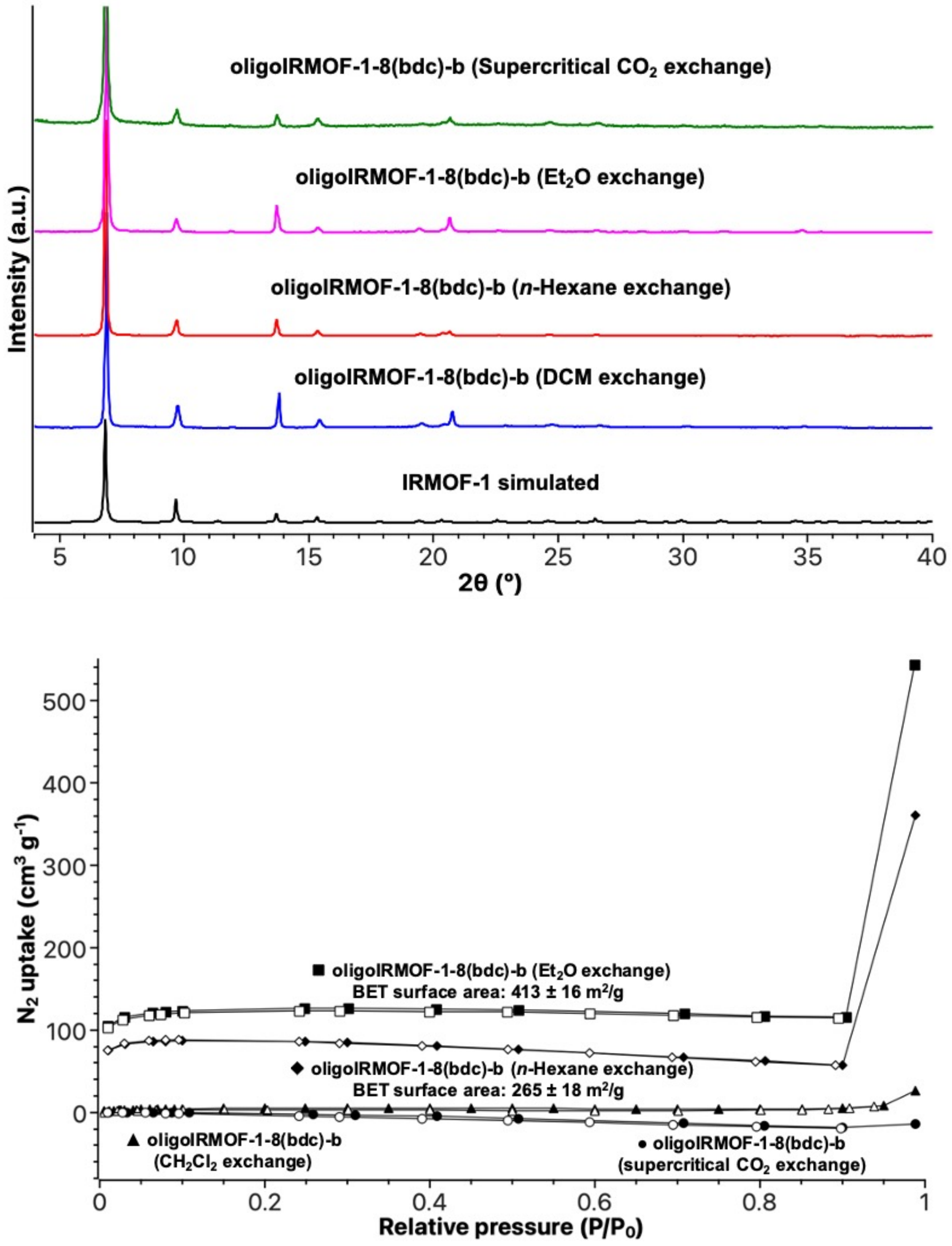

Figure S10. *Top*: PXRD patterns of oligoIRMOF-1-8(bdc)-b after different activation conditions: CH_2Cl_2, *n*-hexane, Et_2O, and supercritical CO_2 exchanged. *Bottom*: N_2 isotherms obtained for oligoIRMOF-1-8(bdc)-b after different activation conditions.

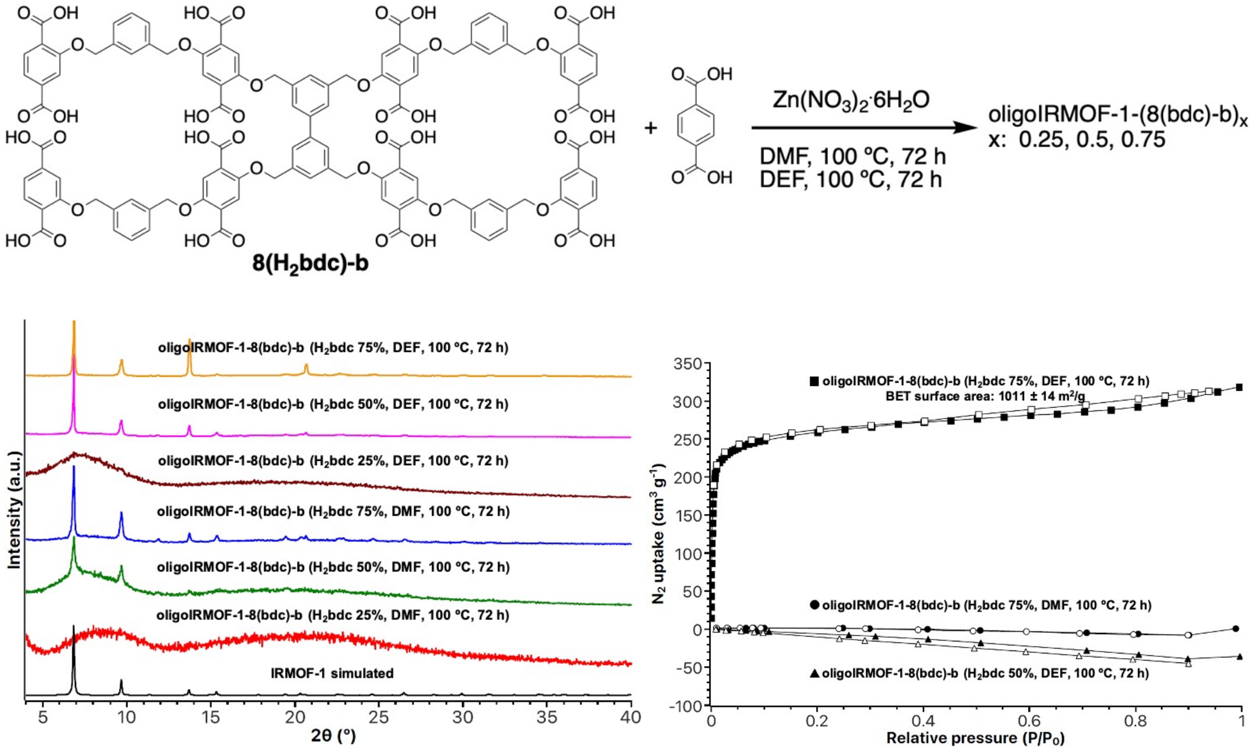

Figure S11. *Top*: Synthetic scheme for mixed-ligand oligoIRMOF-1-8(bdc)-b under different solvent and temperature conditions. *Bottom Left*: PXRD patterns of oligoIRMOF-1-(8(bdc)-b)$_x$ (x = 0.25, 0.5, 0.75) synthesized using different amounts of H_2bdc. *Bottom Right*: N_2 adsorption isotherms of mixed oligoIRMOF-1-(8(bdc)-b)$_x$ (x = 0.25, 0.5, 0.75). The filled and unfilled symbols are corresponding to adsorption and desorption, respectively.

PXRD patterns of IRMOF-1-4(bdc)-l under different conditions

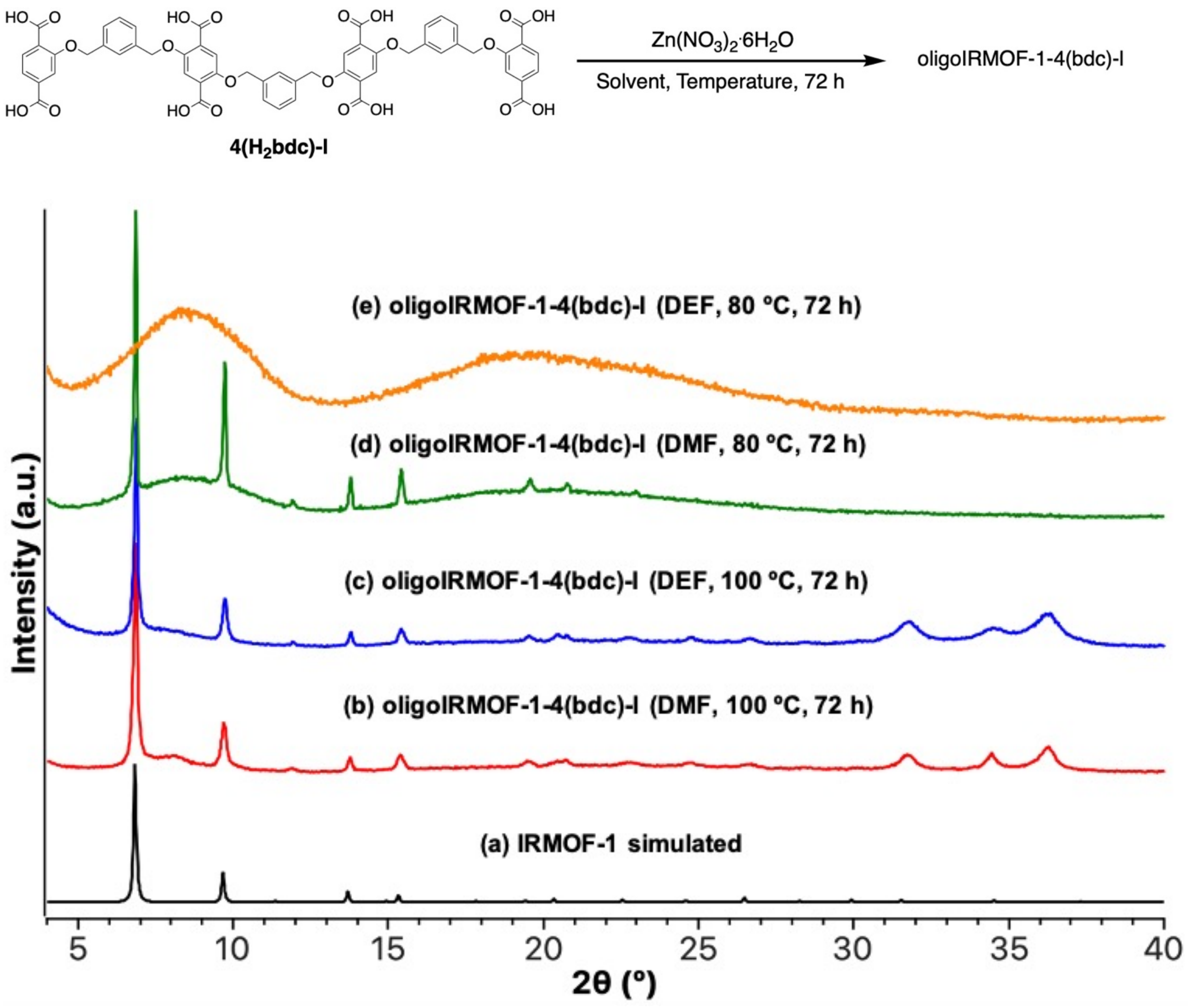

Figure S12. *Top*: Synthetic scheme for oligoIRMOF-1-4(bdc)-l. *Bottom*: PXRD patterns of (a) simulated IRMOF-1, oligoIRMOF-1-4(bdc)-l prepared under different conditions (b) DMF, 100 ºC, 72 h, (c) DEF, 100 ºC, 72 h, (d) DMF, 80 ºC, 72 h, and (e) DEF, 80 ºC, 72 h.

^{1}H and ^{13}C NMR spectrum of oligomeric ligands

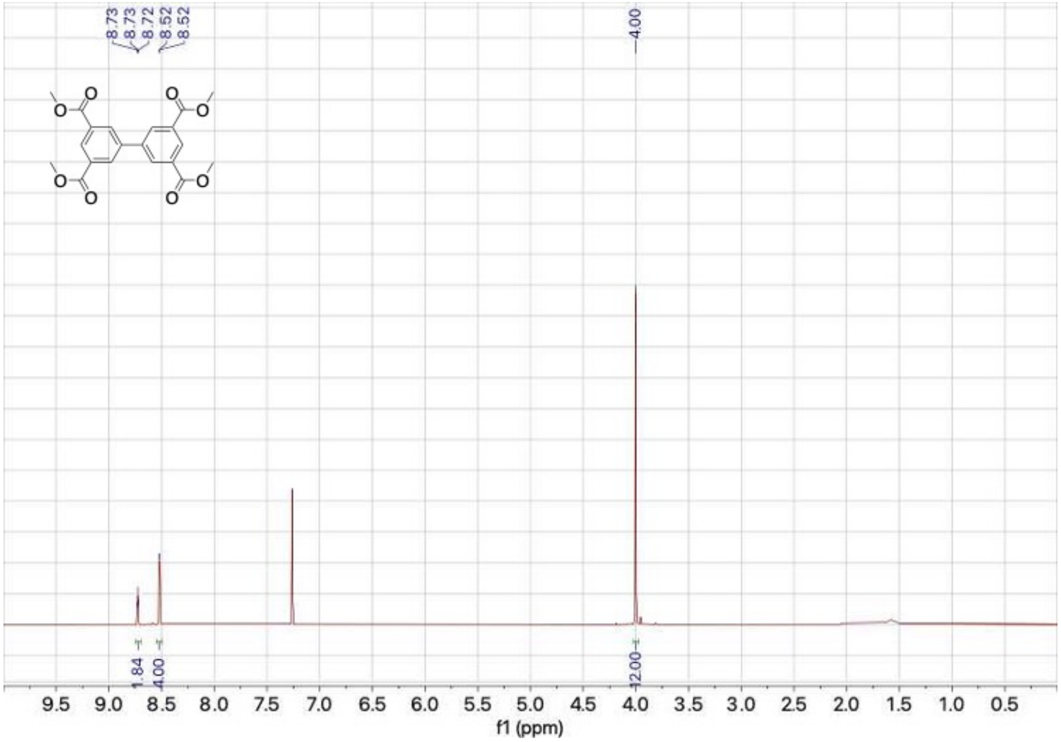

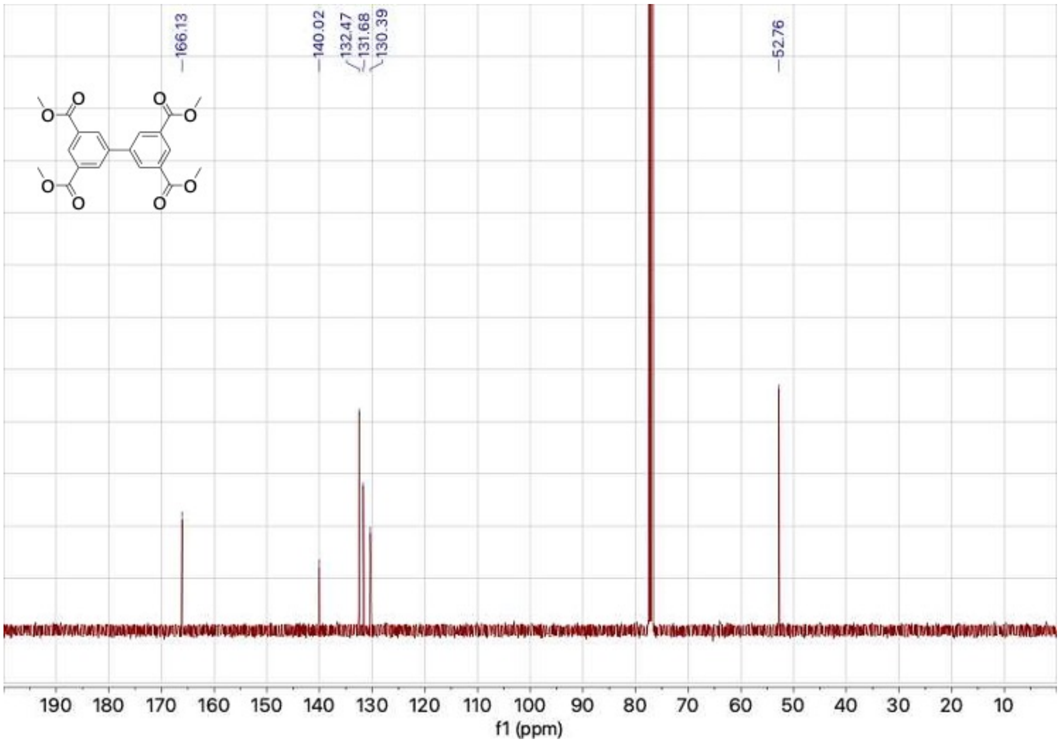

Figure S13. ^{1}H (*top*) and ^{13}C (*bottom*) NMR spectrum of 3,3',5,5'-tetrakis(methoxycarbonyl)biphenyl (**2**).

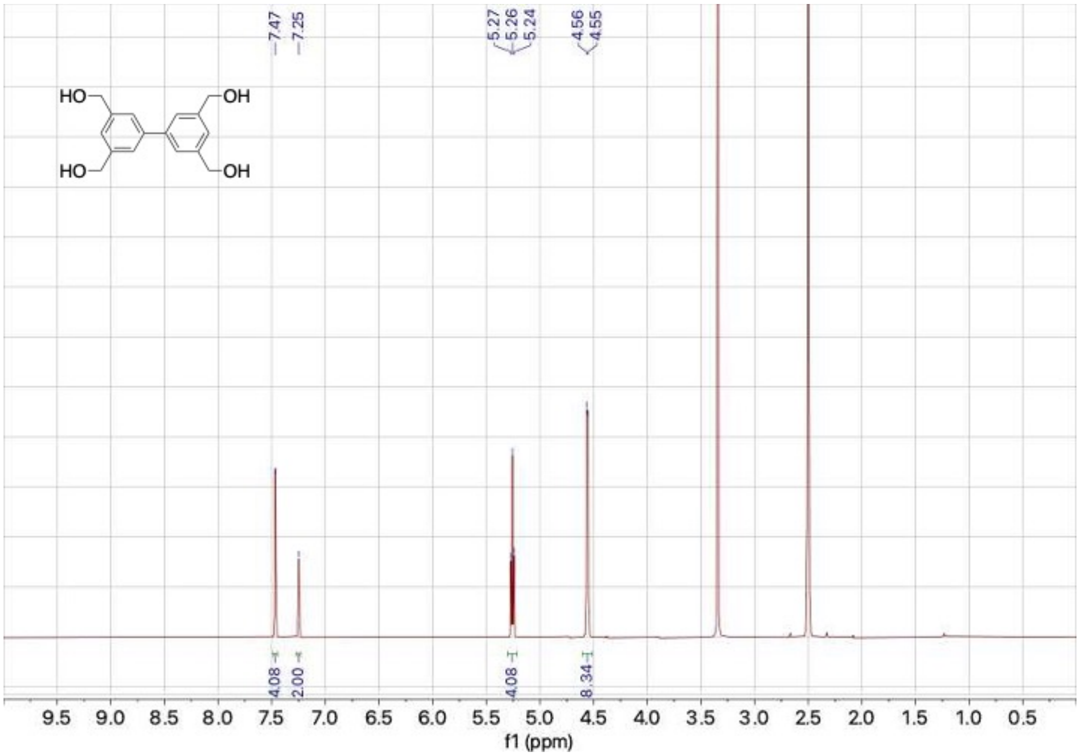

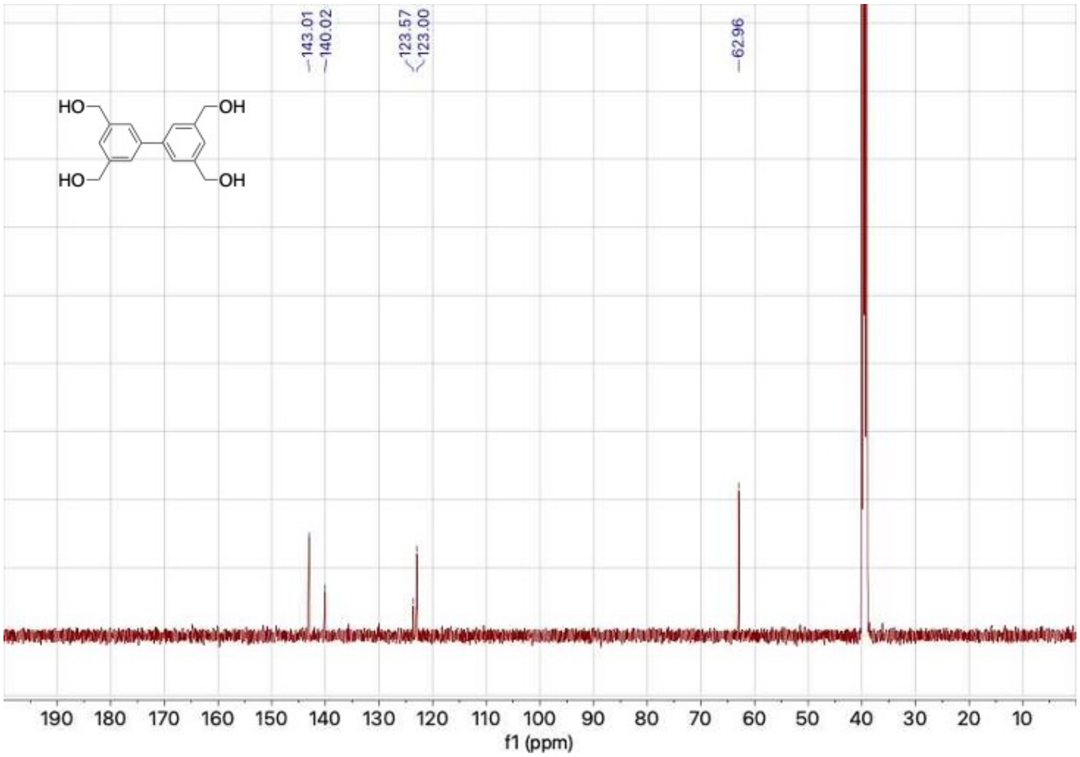

Figure S14. ^{1}H (*top*) and ^{13}C (*bottom*) NMR spectrum of [1,1'-biphenyl]-3,3',5,5'-tetrayltetramethanol (**3**).

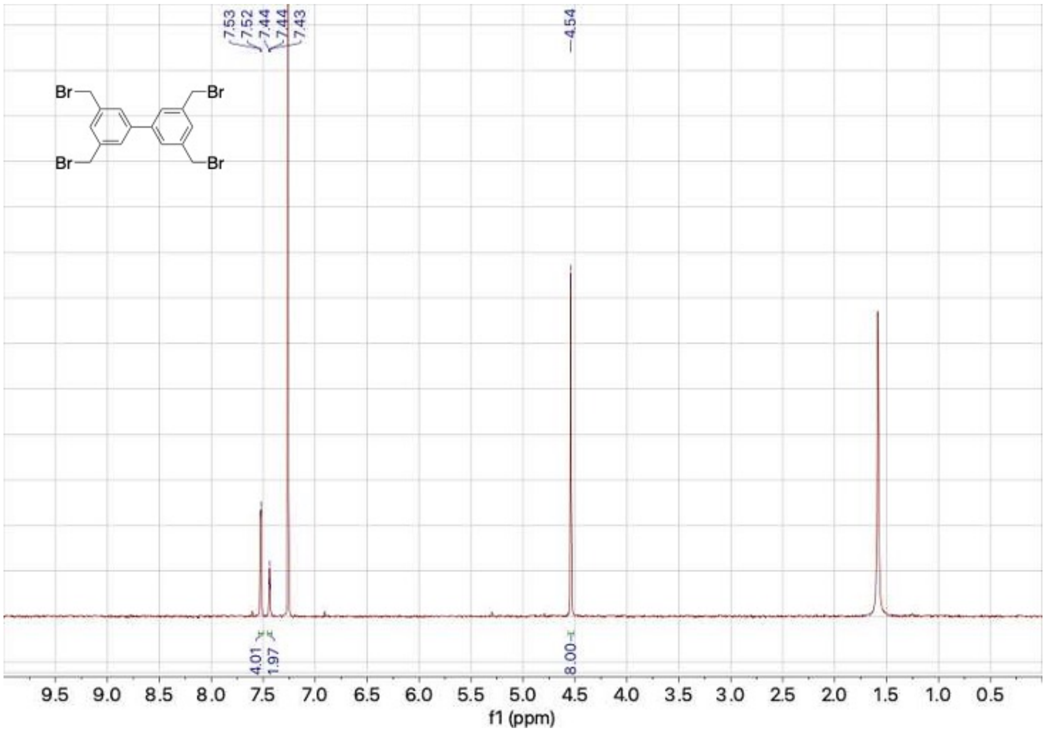

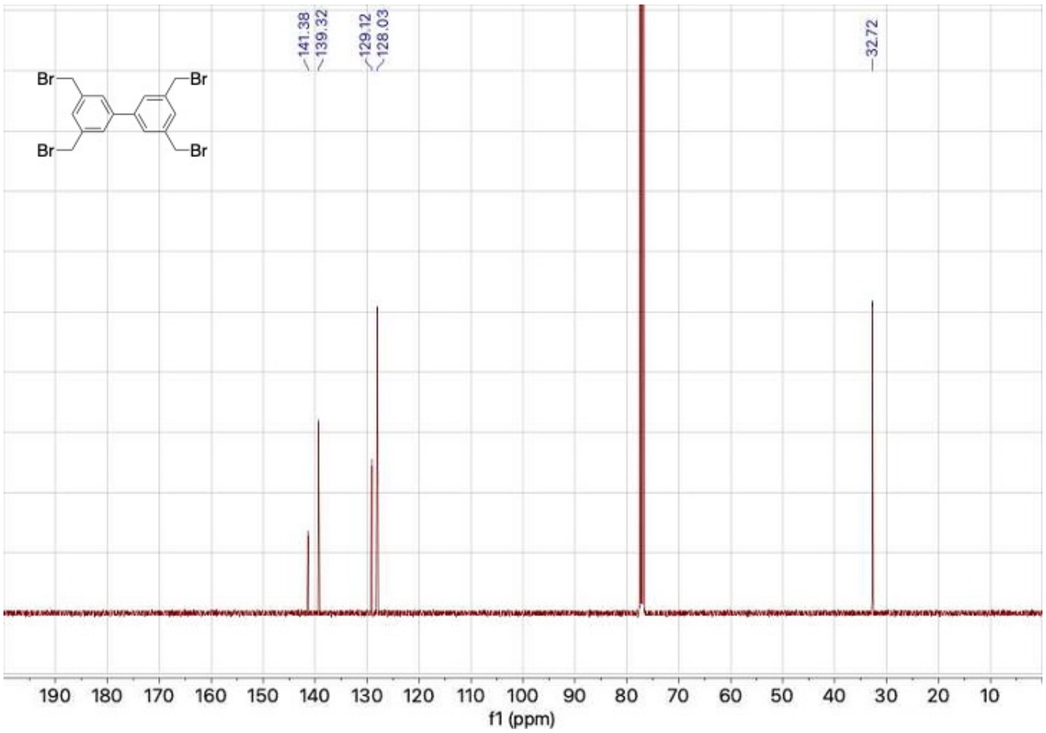

Figure S15. ^{1}H (*top*) and ^{13}C (*bottom*) NMR spectrum of 3,3',5,5'-tetrakis(bromomethyl)-1,1'-biphenyl (**4**).

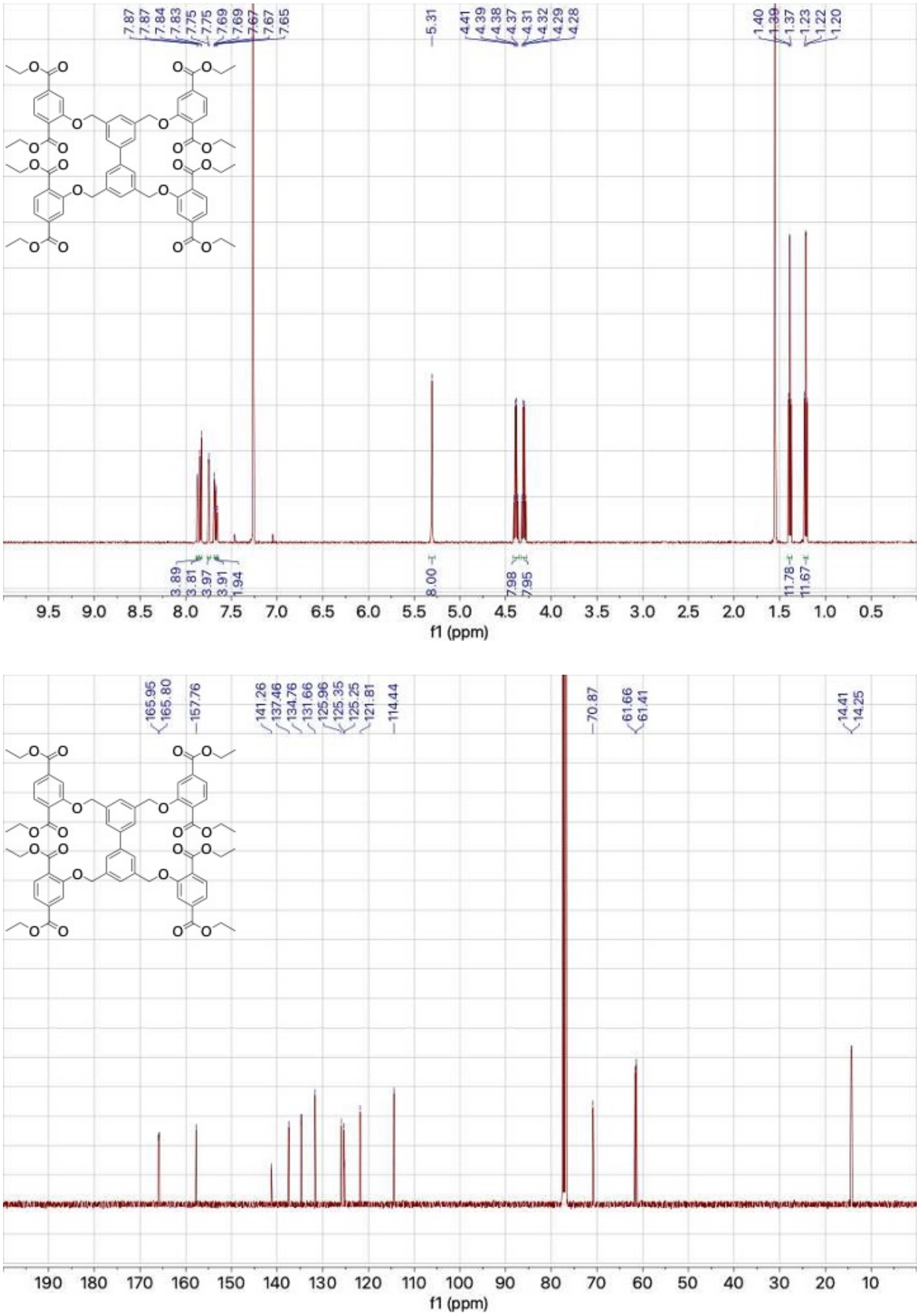

Figure S16. ^{1}H (*top*) and ^{13}C (*bottom*) NMR spectrum of octaethyl 2,2',2'',2'''-(([1,1'-biphenyl]-3,3',5,5'-tetrayltetrakis(methylene))tetrakis(oxy))tetraterephthalate (**6**).

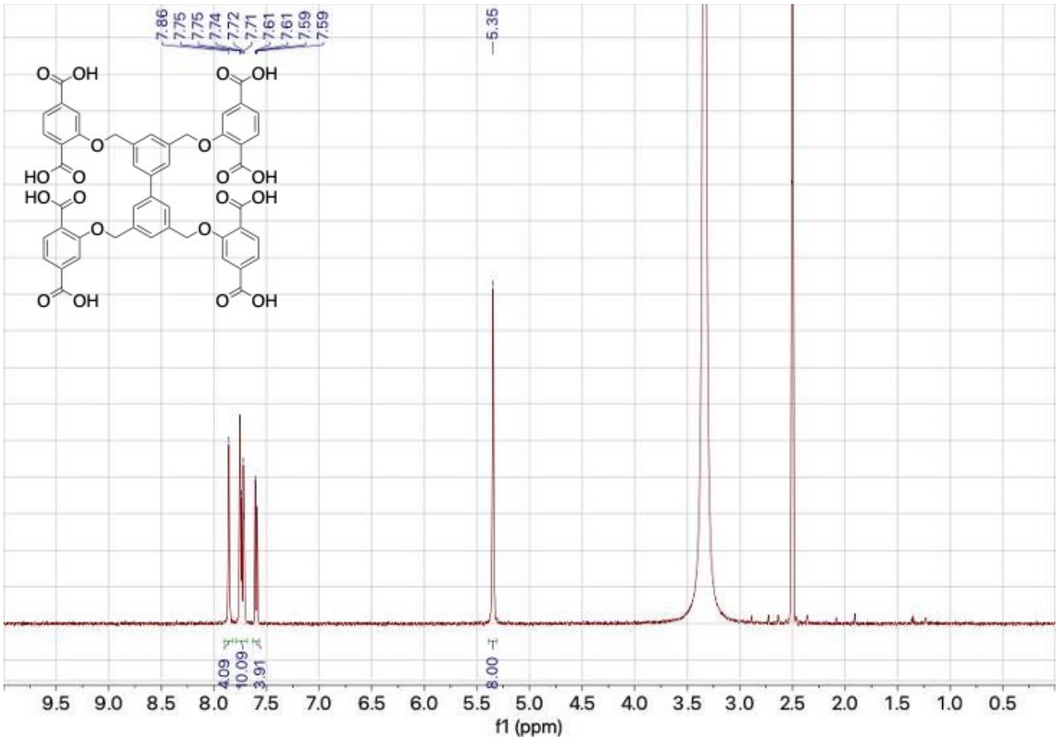

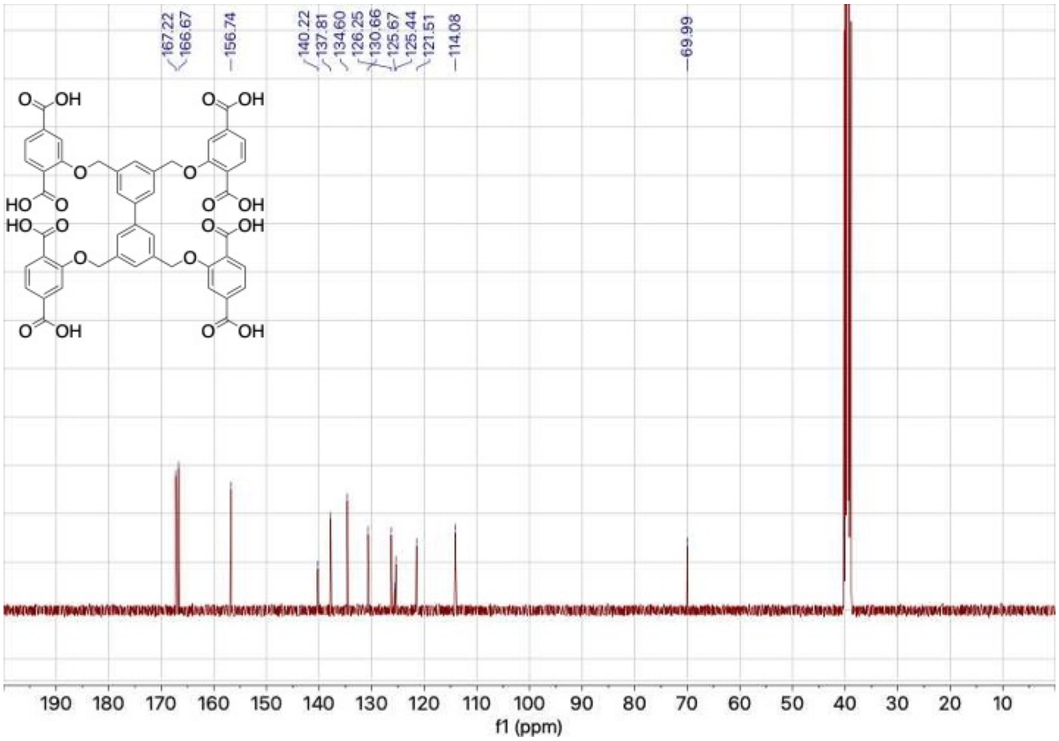

Figure S17. ^{1}H (*top*) and ^{13}C (*bottom*) NMR spectrum of **4(H_2bdc)-b**.

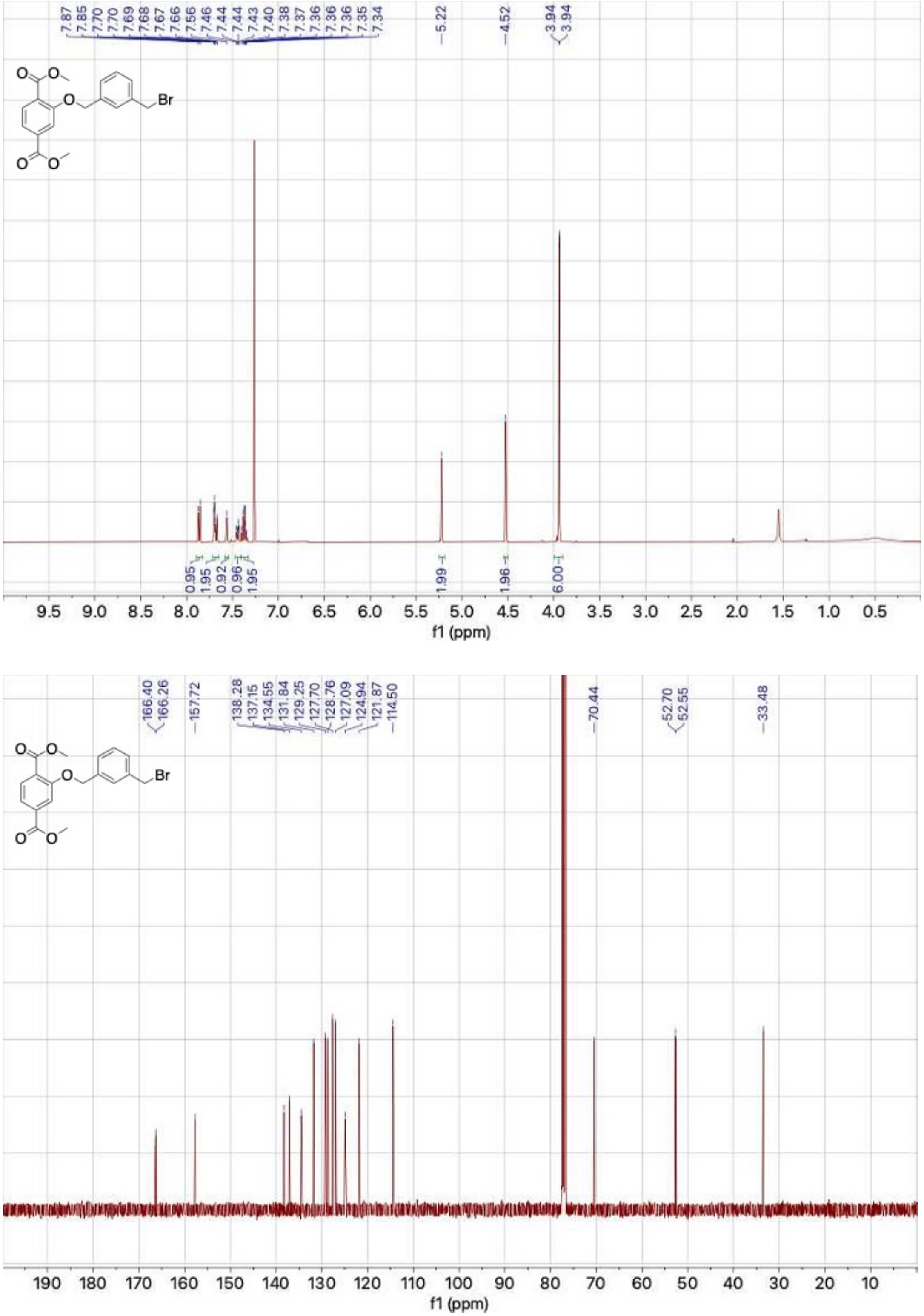

Figure S18. ^{1}H (*top*) and ^{13}C (*bottom*) NMR spectrum of dimethyl 2-((3-(bromomethyl)benzyl)oxy)terephthalate **(9)**.

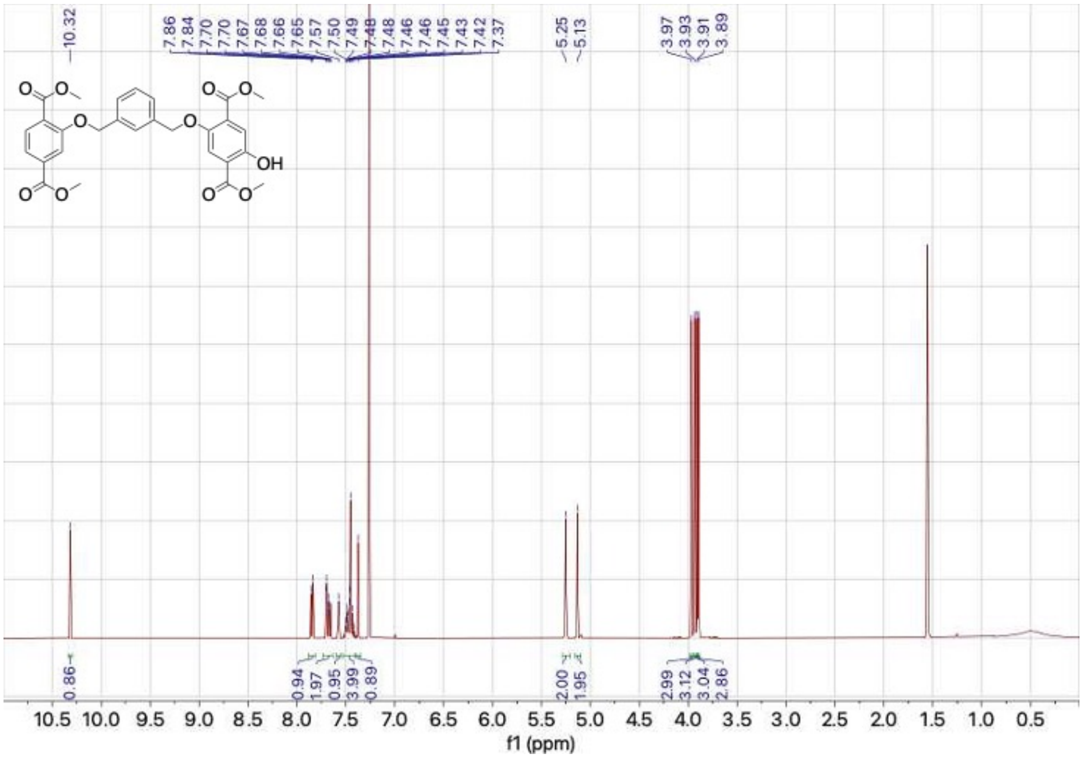

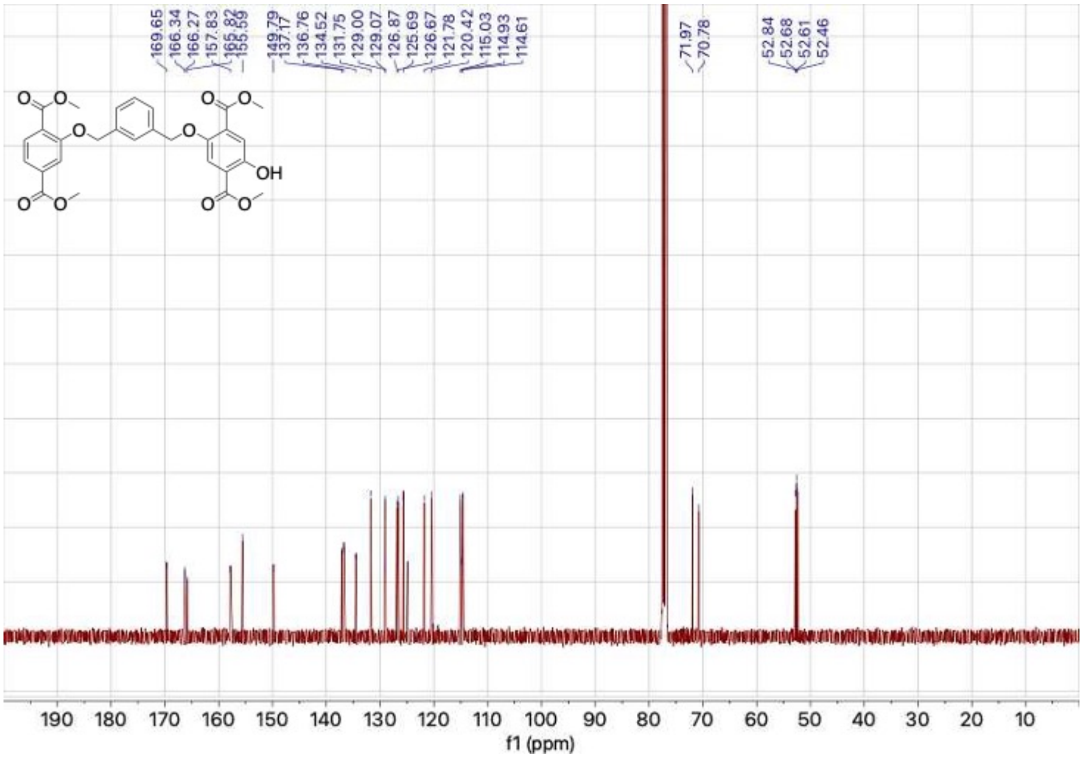

Figure S19. ^{1}H (*top*) and ^{13}C (*bottom*) NMR spectrum of dimethyl 2-((3-((2,5-bis(methoxycarbonyl)phenoxy)methyl)benzyl)oxy)-5-hydroxyterephthalate (**10**).

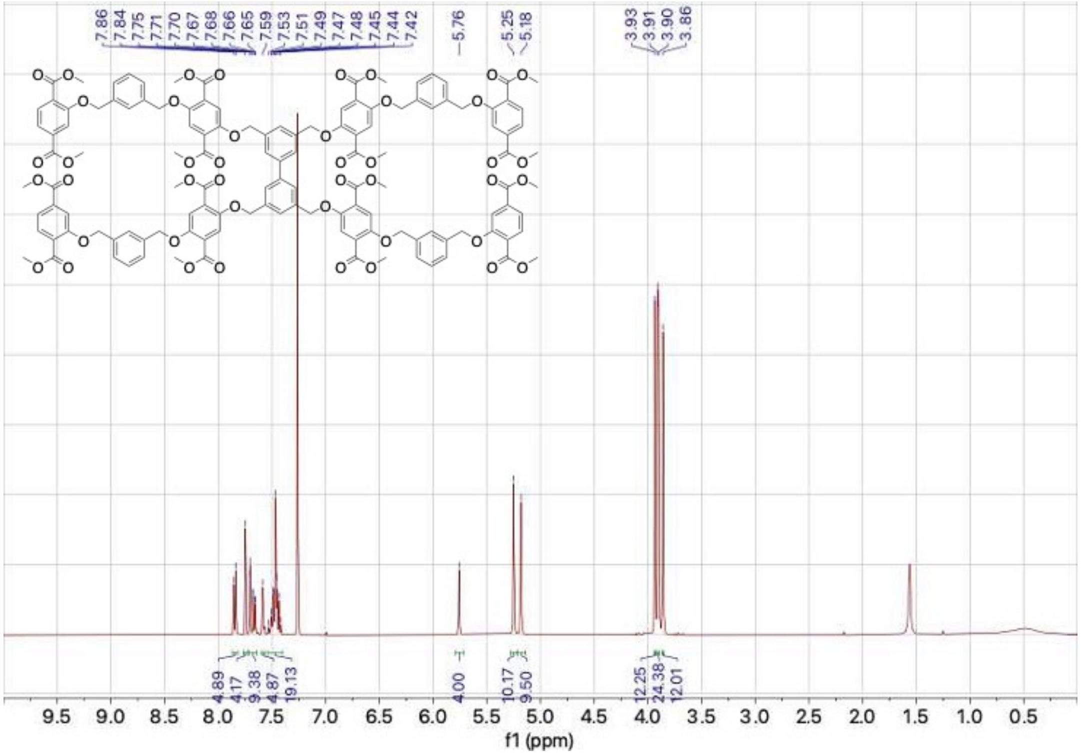

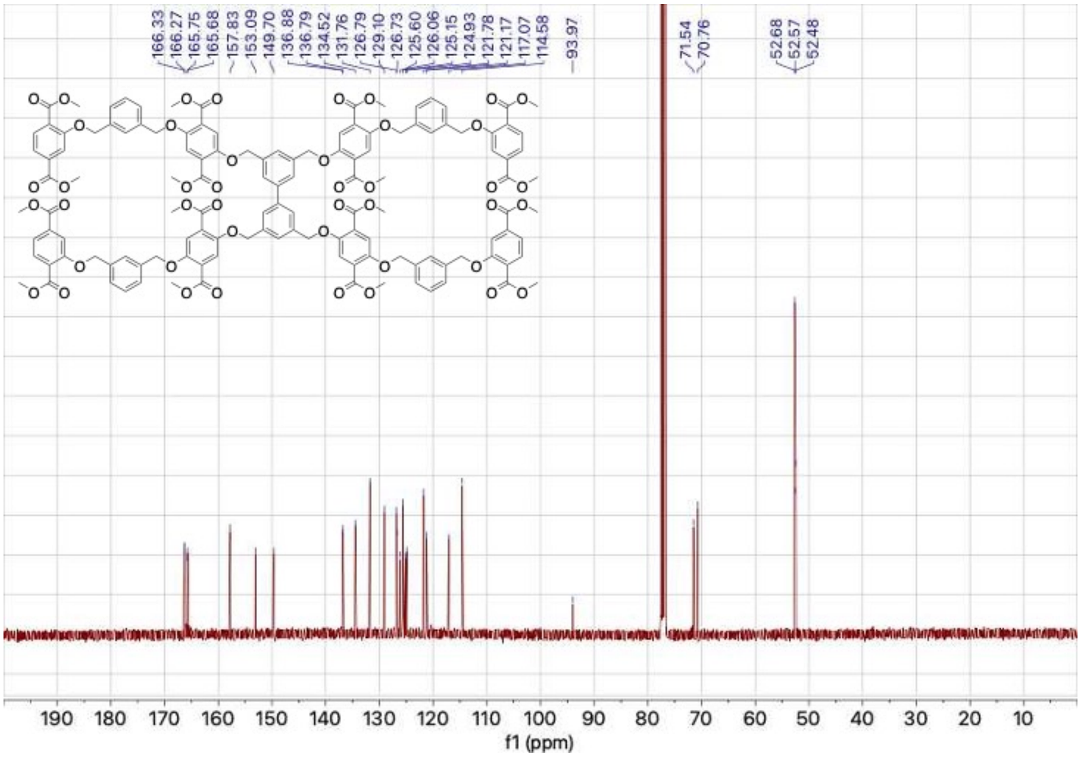

Figure S20. ^{1}H (*top*) and ^{13}C (*bottom*) NMR spectrum of octamethyl 5,5',5'',5'''-(([1,1'-biphenyl]-3,3',5,5'-tetrayltetrakis(methylene))tetrakis(oxy))tetrakis(2-((3-((2,5-bis(methoxycarbonyl)phenoxy)methyl)benzyl)oxy)terephthalate) (**11**).

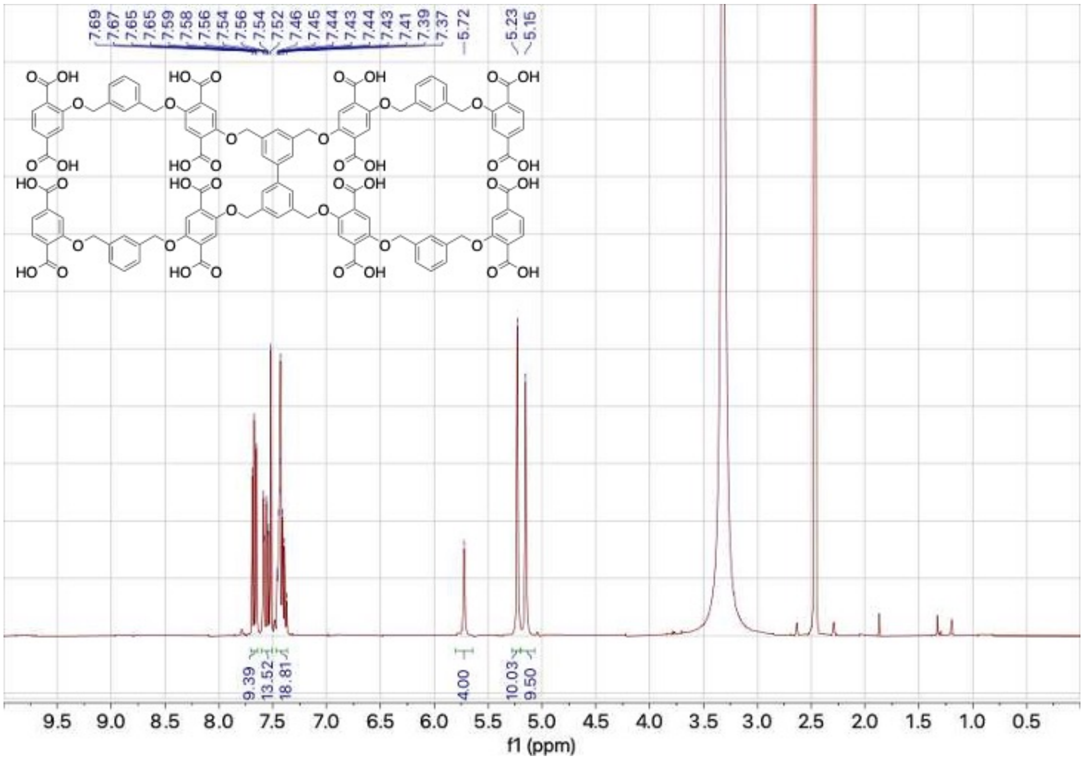

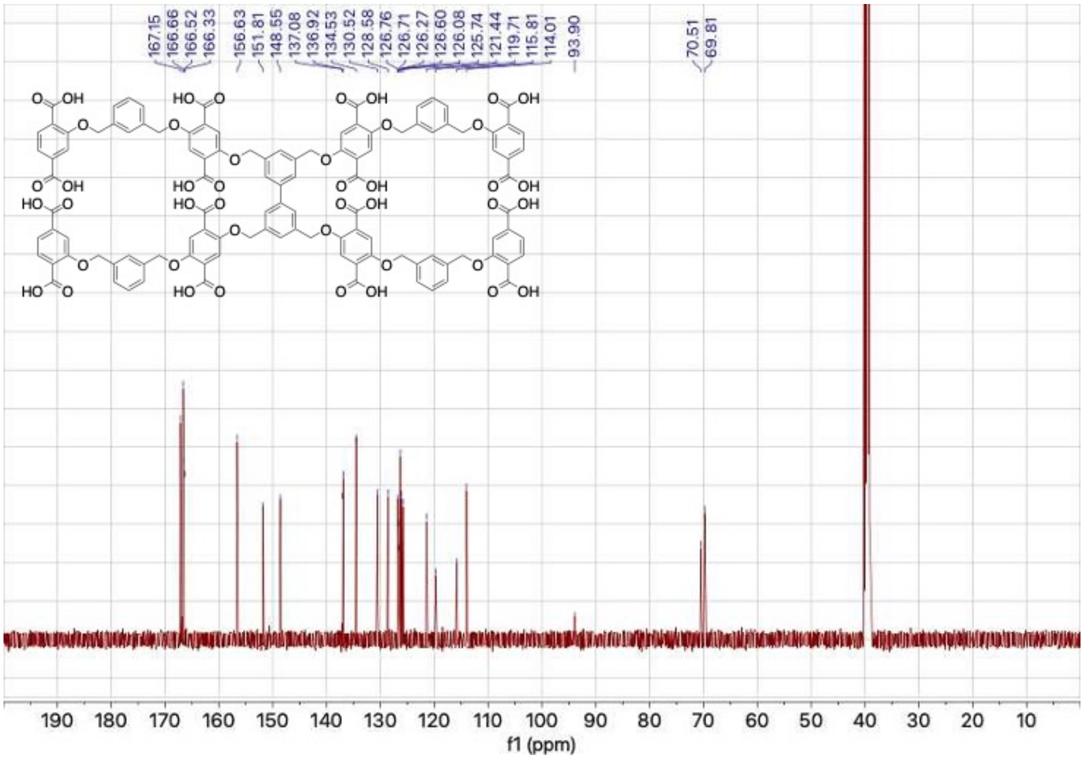

Figure S21. ^{1}H (*top*) and ^{13}C (*bottom*) NMR spectrum of **8(H_2bdc)-b**.

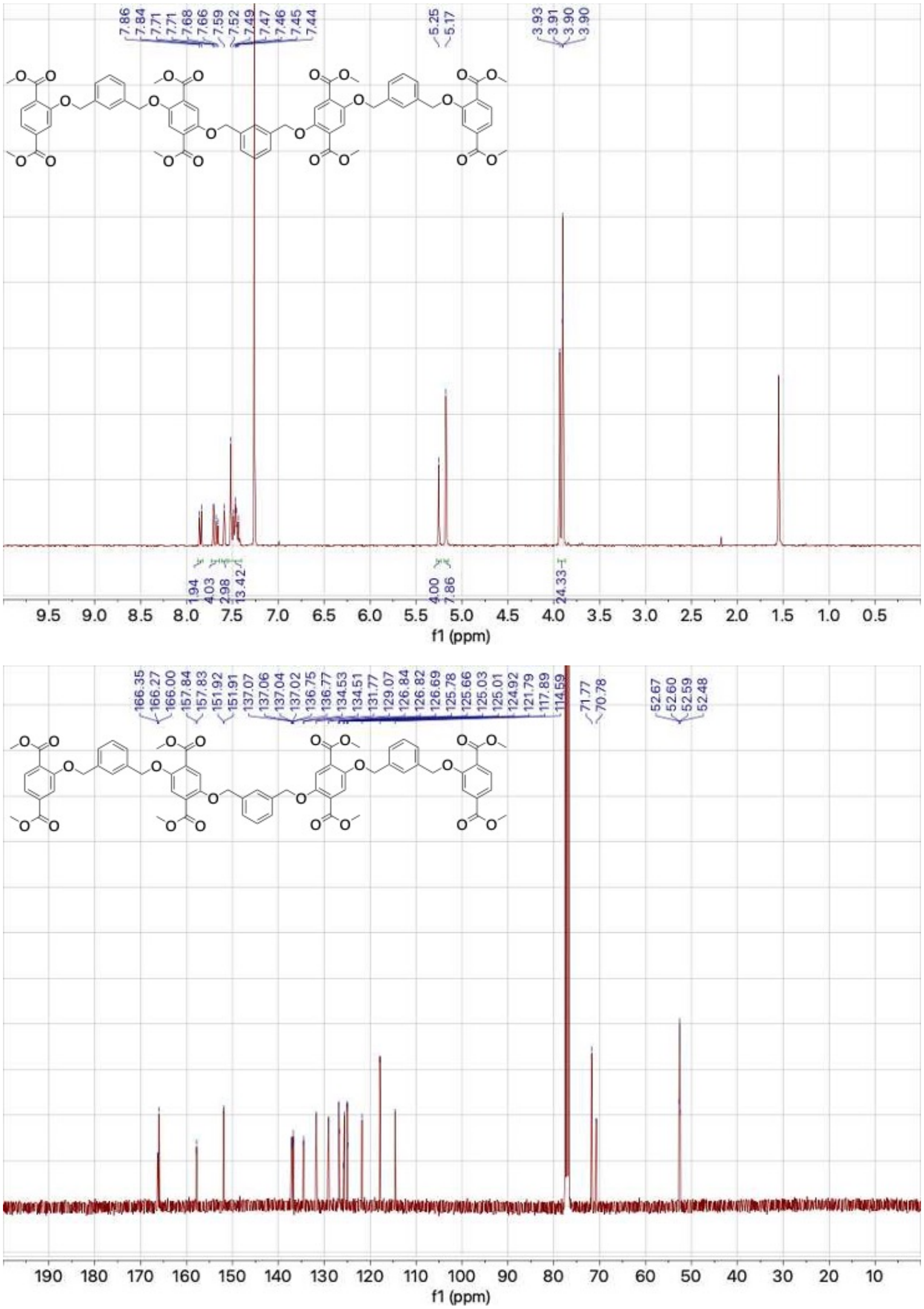

Figure S22. ^{1}H (*top*) and ^{13}C (*bottom*) NMR spectrum of tetramethyl 5,5'-((1,3-phenylenebis(methylene))bis(oxy))bis(2-((3-((2,5-bis(methoxycarbonyl)phenoxy)methyl)benzyl)oxy)terephthalate) **(12)**.

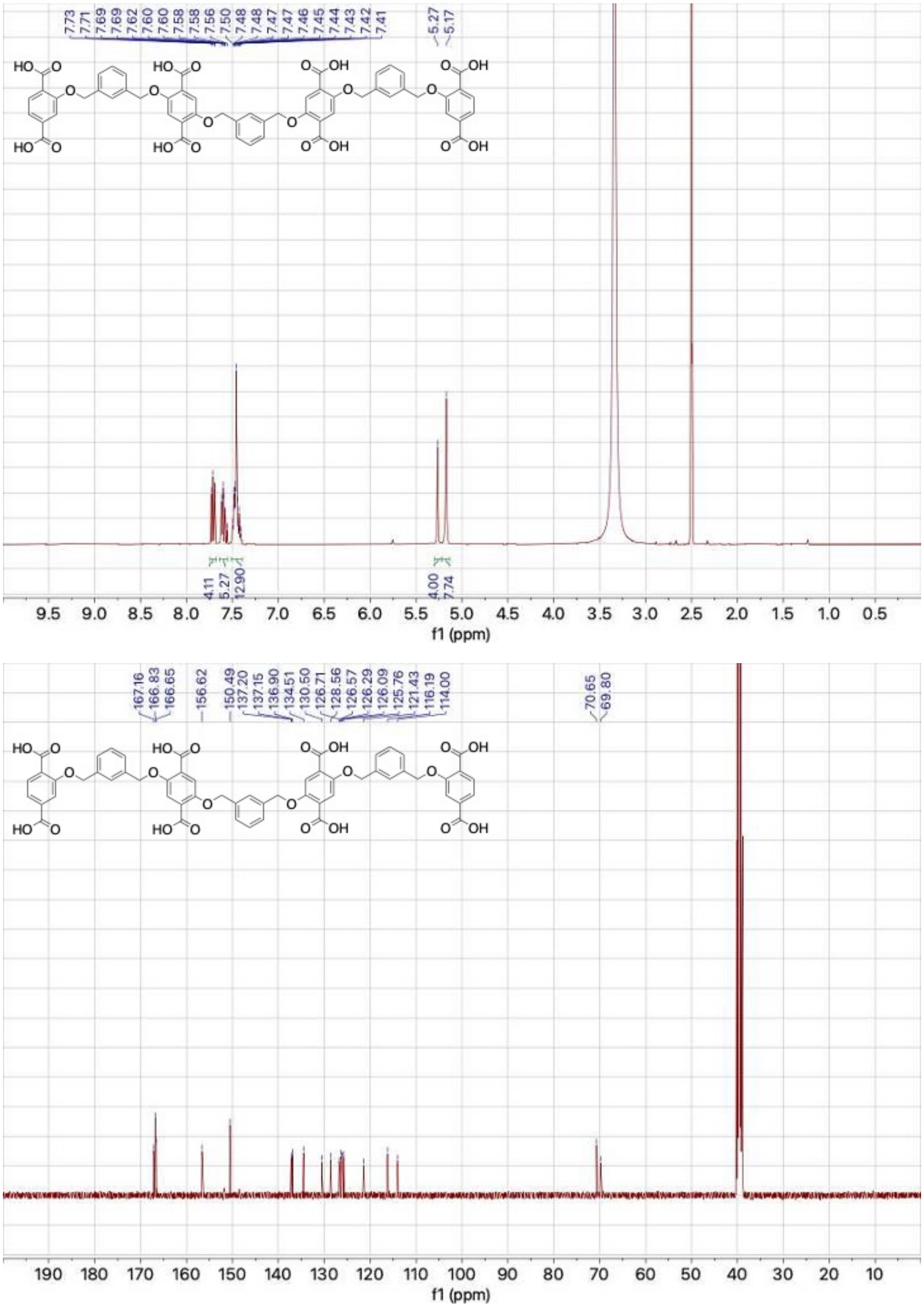

Figure S23. ^{1}H (*top*) and ^{13}C (*bottom*) NMR spectrum of **4(H_2bdc)-l**.

^{1}H NMR spectrum of digested oligoMOFs

Around 3 mg of activated samples were digested in the mixture of 600 µL of DMSO-d_6 and 40 µL of 35 % DCl in D_2O. The resulting solutions were analyzed by ^{1}H NMR.

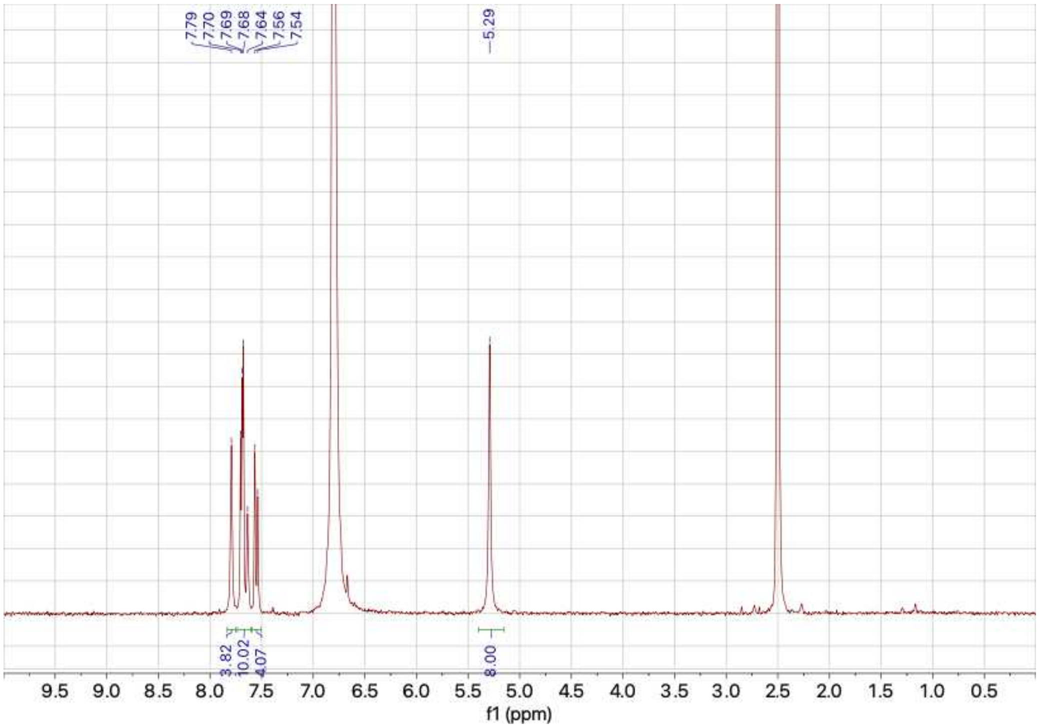

Figure S24. ^{1}H NMR spectrum of digested oligoIRMOF-1-4(bdc)-b.

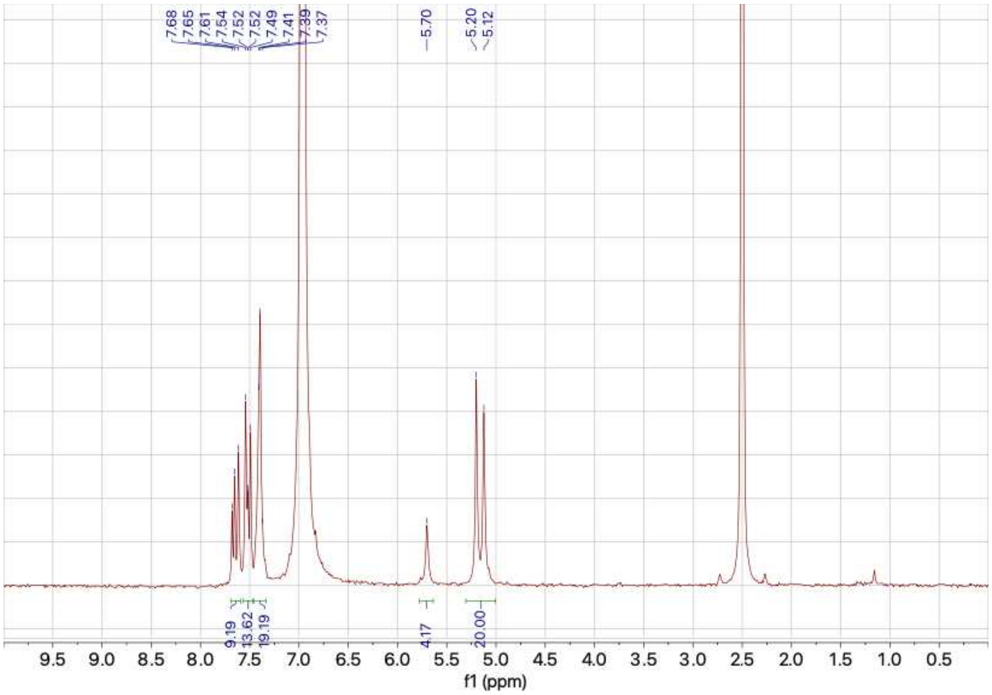

Figure S25. ^{1}H NMR spectrum of digested oligoIRMOF-1-8(bdc)-b.

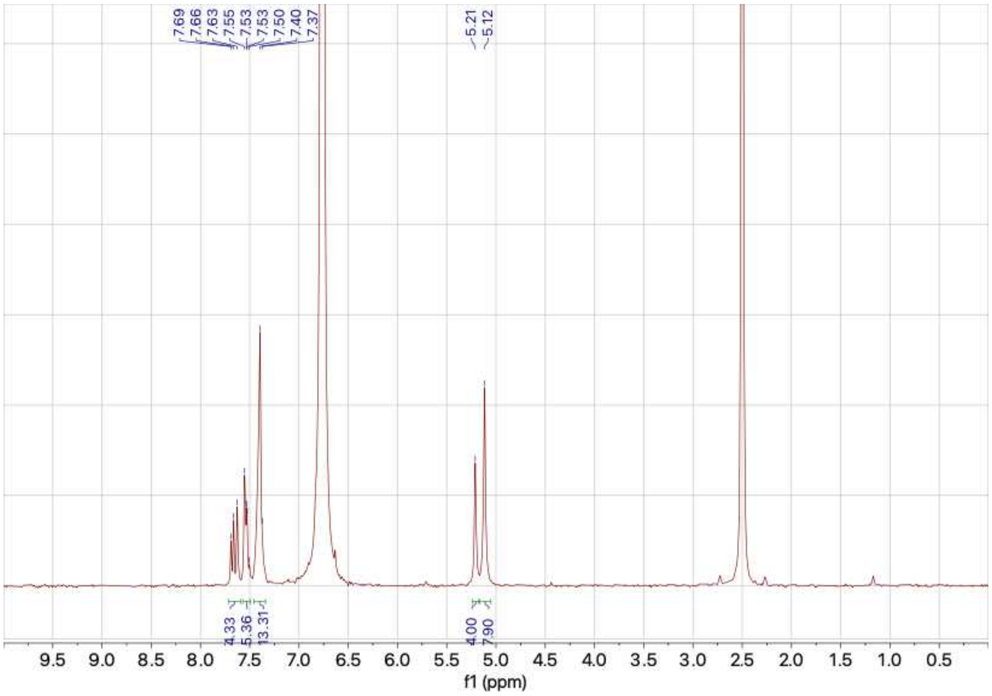

Figure S26. ^{1}H NMR spectrum of digested oligoIRMOF-1-4(bdc)-l.

Inductively coupled plasma mass spectrometry (ICP-MS) analysis of digested oligoMOFs

Approximately 2-3 mg of activated oligoMOF samples were digested in a mixture of nitric acid (70%, 700 µL) and hydrogen peroxide (30%, 300 µL). The resulting clear solutions were diluted to a volume of 100 mL with deionized water. An aliquot of the solution (1 mL) was further diluted to 10 mL with deionized water. The diluted solutions were analyzed using a Thermo iCAP RQ ICP-MS instrument to determine the zinc content in the digested samples.

To calculate the zinc content in the digested sample solutions, the following equations were utilized:

$$Sample\ zinc\ content\ (mass\ \%) = \frac{Zinc\ content\ from\ ICP\ MS\ (\mu g/L)}{Sample\ concentration\ (\mu g/L)} \times 100$$

$$Missing\ zinc\ content\ (\%) = \frac{Theoretical\ Zn\ (\mu g/L)\ -\ Measured\ Zn\ (\mu g/L)}{Theoretical\ Zn\ (\mu g/L)} \times 100$$

For calculating the theoretical zinc content of each oligoMOF, the following molecular formulas were utilized: oligoIRMOF-1-4(bdc)-b = Zn_4O(**4(bdc)-b**)$_{0.75}$, oligoIRMOF-1-4(bdc)-l = Zn_4O(**4(bdc)-l**)$_{0.75}$, and oligoIRMOF-1-8(bdc)-b = Zn_4O(**8(bdc)-b**)$_{0.375}$). The corresponding molecular weights for each of these oligoMOFs is listed in Table S1.

Table S1. Observed zinc content determined by ICP-MS from digested, diluted oligoMOF samples, calculated M:L ratio, and unreacted zinc contents.

MOF	Sample Amount (mg)	OligoMOF MW (g/mol)	Ligand MW (g/mol)	Measured Zinc (µg/L)	Theoretical Zinc (ug/L)	% Experimental vs. Theoretical Zn Content	% Missing Zinc from Theoretical
oligoIRMOF-1-4(bdc)-b	3.45	975.60	930.78	780	925	84.3	15.7
oligoIRMOF-1-4(bdc)-l	2.65	1077.72	1066.93	500	643	77.8	22.2
oligoIRMOF-1-8(bdc)-b	3.35	1076.96	2131.84	576	814	70.8	29.2

Scanning Electron Microscopy (SEM) images of oligoMOFs

MOF samples were dispersed in CH_2Cl_2 (~1 mg/mL) and placed them onto silicon wafers using a thin glass capillary. The silicon wafers were mounted on an aluminum sample holder disk with carbon tape and coated using an Ir-sputter coating for 60 sec. A FEI Quanta FEG 250 SEM instrument was used for acquiring images using an accelerating voltage of 5 kV under vacuum at a working distance at 10 mm.

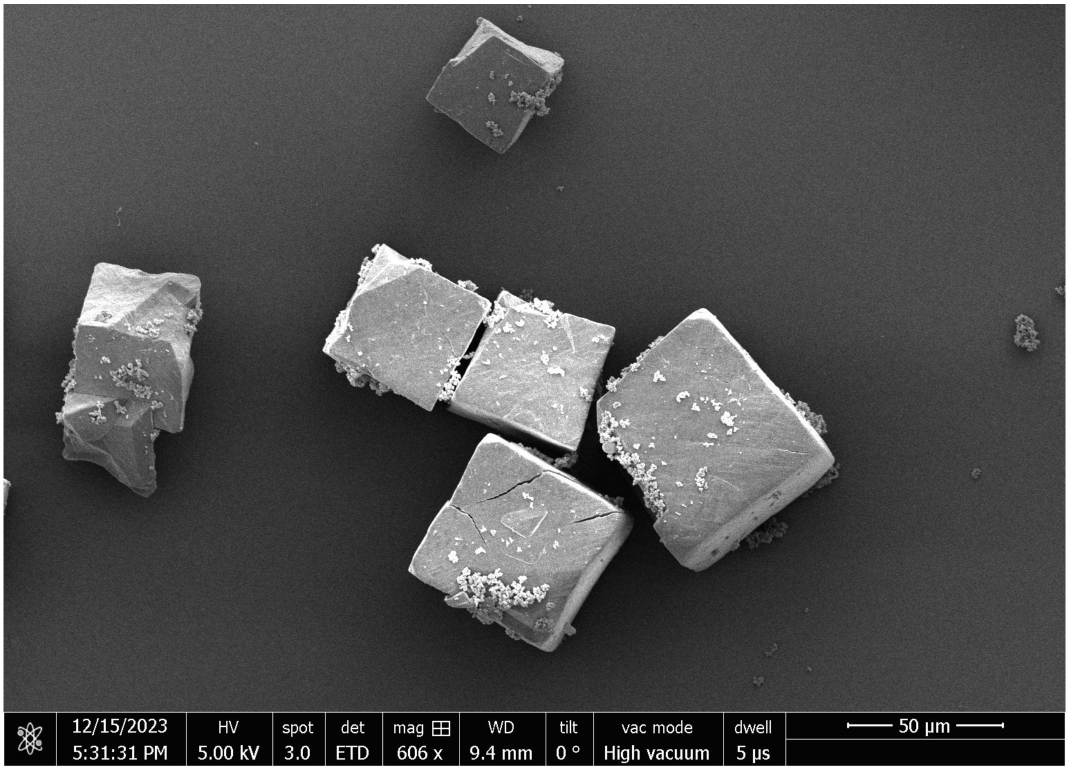

Figure S27. SEM image of oligoIRMOF-1-4(bdc)-b.

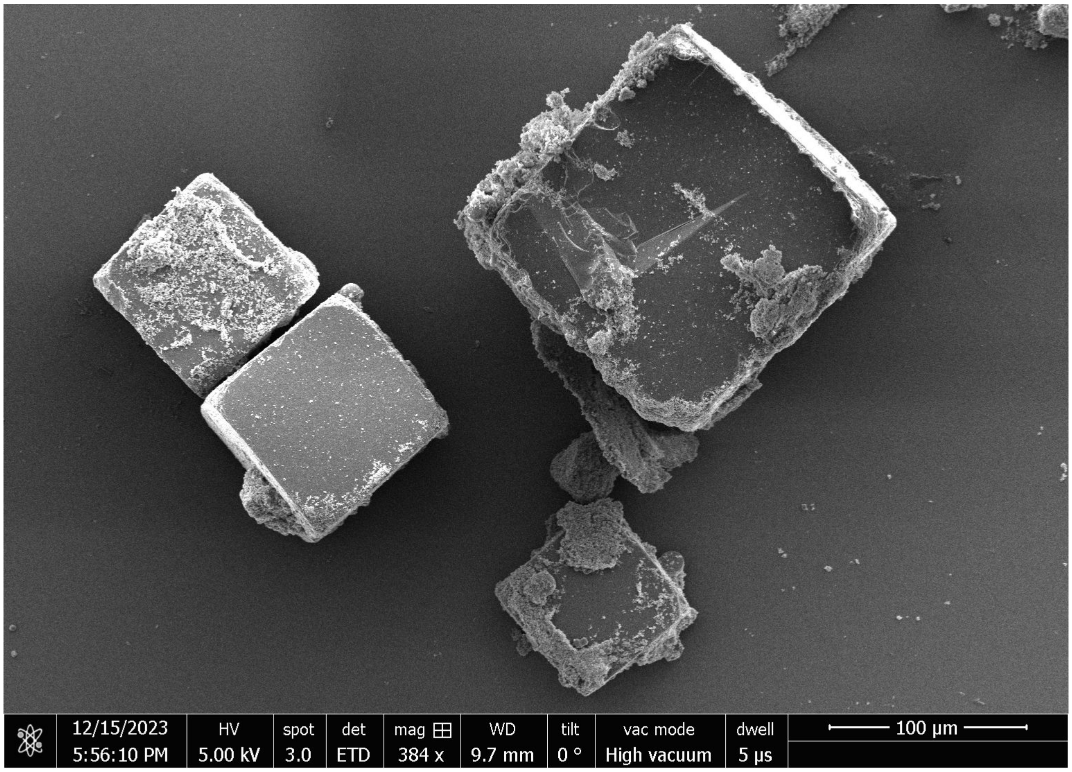

Figure S28. SEM image of oligoIRMOF-1-8(bdc)-b.

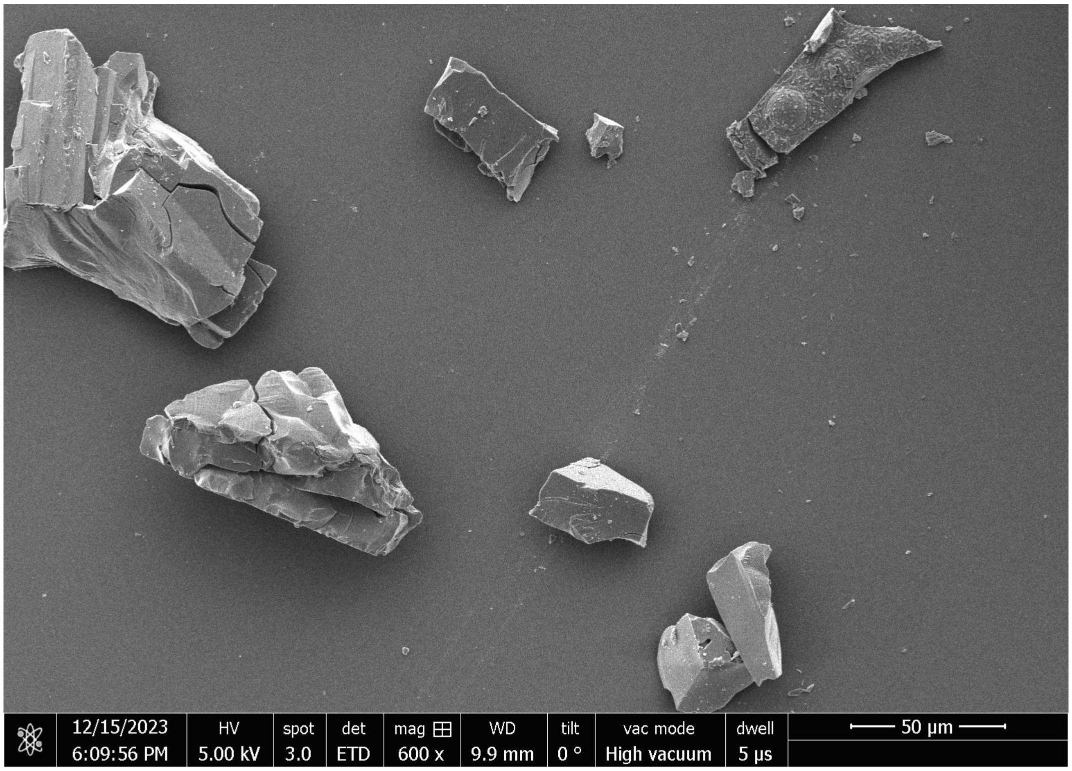

Figure S29. SEM image of oligoIRMOF-1-4(bdc)-l.
